# Prognostic value and immune landscapes of immunogenic cell death-associated lncRNAs in lung adenocarcinoma

**DOI:** 10.1038/s41598-023-46669-w

**Published:** 2023-11-06

**Authors:** Kexin Shu, Chenxi Cai, Wanying Chen, Jiatong Ding, Zishun Guo, Yiping Wei, Wenxiong Zhang

**Affiliations:** 1 Department of Thoracic Surgery, The Second Affiliated Hospital of Nanchang University, 1 Minde Road, Nanchang, 330006 China; 2 Jiangxi Medical College, Nanchang University, Nanchang, 330006 China

**Keywords:** Cancer, Computational biology and bioinformatics, Genetics, Immunology

## Abstract

Immunogenic cell death (ICD) has been demonstrated to activate T cells to kill tumor cells, which is closely related to tumor development, and long noncoding RNAs (lncRNAs) are also involved. However, it is not known whether ICD-related lncRNAs are associated with the development of lung adenocarcinoma (LUAD). We downloaded ICD-related genes from GeneCards and the transcriptome statistics of LUAD patients from The Cancer Genome Atlas (TCGA) and subsequently developed and verified a predictive model. A successful model was used together with other clinical features to construct a nomogram for predicting patient survival. To further study the mechanism of tumor action and to guide therapy, we performed enrichment analysis, tumor microenvironment analysis, somatic mutation analysis, drug sensitivity analysis and real-time quantitative polymerase chain reaction (RT-qPCR) analysis. Nine ICD-related lncRNAs with significant prognostic relevance were selected for model construction. Survival analysis demonstrated that overall survival was substantially shorter in the high-risk group than in the low-risk group (P < 0.001). This model was predictive of prognosis across all clinical subgroups. Cox regression analysis further supported the independent prediction ability of the model. Ultimately, a nomogram depending on stage and risk score was created and showed a better predictive performance than the nomogram without the risk score. Through enrichment analysis, the enriched pathways in the high-risk group were found to be primarily associated with metabolism and DNA replication. Tumor microenvironment analysis suggested that the immune cell concentration was lower in the high-risk group. Somatic mutation analysis revealed that the high-risk group contained more tumor mutations (P = 0.00018). Tumor immune dysfunction and exclusion scores exhibited greater sensitivity to immunotherapy in the high-risk group (P < 0.001). Drug sensitivity analysis suggested that the predictive model can also be applied to the choice of chemotherapy drugs. RT-qPCR analysis also validated the accuracy of the constructed model based on nine ICD-related lncRNAs. The prognostic model constructed based on the nine ICD-related lncRNAs showed good application value in assessing prognosis and guiding clinical therapy.

## Introduction

Accounting for 21% of all cancer deaths globally, lung cancer (LC) has become the primary cause of mortality^1^. The most common subtype of LC is lung adenocarcinoma (LUAD), and the morbidity and mortality of LUAD continue to increase yearly^2^. Tumor node metastasis (TNM) staging is frequently used to forecast clinical outcomes, but the predictive effect is still unsatisfactory^3,4^. Thus, it is imperative to build a better assessment measure for predicting patient survival and guiding LUAD treatment. In recent years, the approach of constructing a predictive model through a combination of several biomarkers and using it to assess tumor patient prognosis has been widely used^5–7^.

By activating T cells to produce direct impacts on tumor cell killing and antitumor immune responses, immunogenic cell death (ICD) is an example of regulated cell death that can regulate the growth of tumors^8^. Long noncoding RNAs (lncRNAs) can regulate tumor development by affecting tumor cell metabolism and certain oncogenic or carcinogenic factors; for example, cancer-associated fibroblast-specific lncRNA (LINC01614) enhances glutamine uptake in LUAD, thereby promoting cancer cell growth^9^. A variety of models constructed with lncRNAs to predict the prognosis and treatment options for various cancer types are now available and have also shown good prognostic value^5,6,10^. ICD-related lncRNA models have been developed to forecast the development of high-grade gliomas (HGGs), head and neck squamous cell carcinoma (HNSC) and gastric cancer (GC)^11–13^, but there remains a dearth of ICD-related biomarkers for evaluating LUAD prognosis.

In this study, we first used Pearson’s analysis to derive ICD-associated lncRNAs that play a role in LUAD, followed by differential analysis, univariate and multivariate cox analysis, and lasso regression to finally screen nine lncRNAs to construct a prognostic model. After the successful verification of this model, we created a nomogram to estimate the survival time of patients with LUAD. Subsequently, we explored the likely mechanisms by which ICD-associated lncRNAs act in LUAD and provided ideas for options for clinical treatment.

## Materials and methods

### Statistics source

We downloaded the transcriptome statistics for LUAD from The Cancer Genome Atlas (TCGA) (https://portal.gdc.cancer.gov/, until October 29th, 2022), consisting of 59 normal samples and 526 tumor samples (585 samples), including FPKM data and count data. The count data were log2-transformed using the “limma” package^14^. Additionally, for further study, the survival information, clinical information, and simple nucleotide variation (SNP) information for LUAD were retrieved from TCGA. The term “immunogenic cell death” was searched in GeneCards (https://www.genecards.org/, until October 29th, 2022), and we selected 138 ICD-related genes (IRGs) based on correlation scores > 35. All statistical analyses were carried out in accordance with R4.2.1.

### Investigation of ICD-related lncRNAs with differential expression

**Figure 1:**
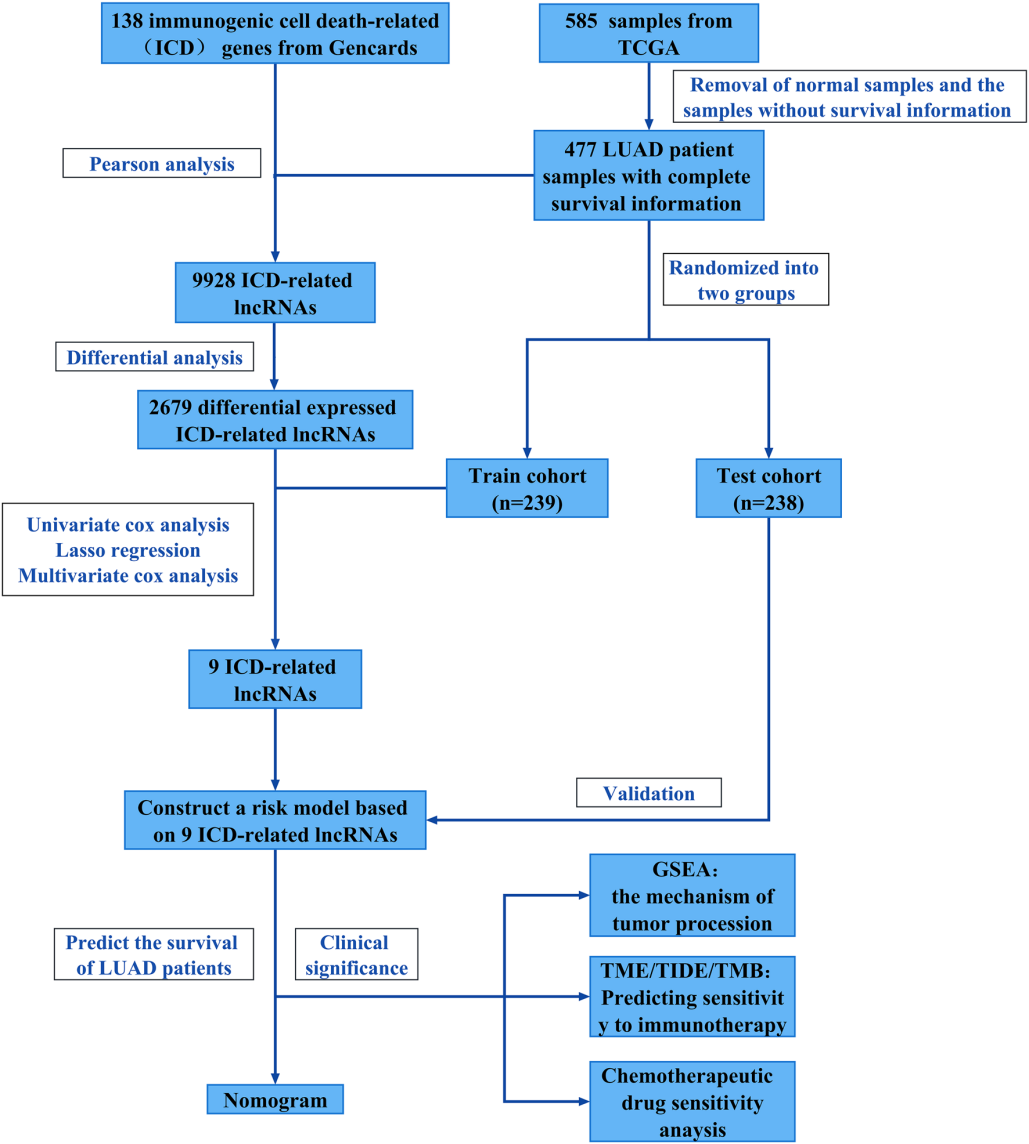
Flowchart.

Our method is displayed in a flowchart (Fig. 1). To identify ICD-related lncRNAs that are differentially expressed, we isolated lncRNAs expressed in LUAD for Pearson analysis with IRGs, selected ICD-related lncRNAs based on P < 0.05 and |correlation coefficient| > 0.3, and then performed difference analysis with the criteria |log2FoldChange| > 1 and P < 0.05.

### Constructing the model

All cases with normal or no survival details were removed, leaving a total of 477 tumor samples. At a random ratio of 1:1, the 477 patients were divided into two groups, with 239 samples in the training subgroup and 238 samples in the test subgroup. The training cohort was used for model construction. To identify ICD-associated lncRNAs that play a major role in LUAD, we carried out univariate Cox analysis to identify ICD-related lncRNAs linked to prognosis (P < 0.05, HR > 1), followed by LASSO analysis to avoid overfitting. Then, we performed multivariate Cox analysis to confirm the prognostic ICD-related lncRNAs to construct the model. RiskScore = EXP_gene1_ * genecoef1 + EXP_gene2_ * genecoef2 +  ⋯ + EXP_genen_ * genecoefn.

### Verifying the model’s feasibility

The test cohort and entire cohort were used for model validation, and employing the RiskScore algorithm, the risk score for each individual was computed. Then, the median of each cohort was used to classify each cohort into high- and low-risk groups. All three cohorts underwent Kaplan‒Meier (K‒M) analysis^15^, receiver operating characteristic (ROC) analysis^16^, risk mapping, heatmapping to visualize expression, and principal component analysis (PCA)^17^ to assess the predictive effect and determine whether there was a discernible distinction in life expectancy between the two risk groups. Stratified analysis can help us understand whether this prognostic model is applicable to the entire population. To confirm whether the constructed model was an independent predictor, we used age, gender, stage, T stage, N stage, and M stage together with the risk score and conducted univariate and multivariate Cox analyses^18^.

### Structuring the nomogram

We investigated the link between the risk score and other clinical factors. To identify whether the risk score has the potential to outperform other clinical indicators as an independent predictor, the predictive effect was compared based on the area under the ROC curve (AUC). Stage and risk score (P < 0.001, HR > 1) were incorporated into a nomogram to predict survival rates at 1 year, 3 years, and 5 years^19^. Two calibration curves were made, one containing risk and stage and one without risk, and then, the fit of the two calibration curves was compared. Decision curve analysis (DCA) was developed to evaluate whether a prognostic model that included a risk score would increase clinical benefit^20^.

### Gene set enrichment analysis (GSEA)

After dividing all samples into two risk groups, we used KEGG, BIOCARTA, PID, GO, REACTOME, and WIKIPATHWAYS in GSEA4.3.2 software to derive different pathways enriched in the two risk groups using P < 0.05 and FDR < 0.25 as criteria to help understand the different mechanisms of LUAD progression and to provide guidance for treatment ideas^21^.

### Evaluation of the tumor immune microenvironment

The stromal cell and immune cell concentrations in the tumors were compared between the two risk groups using the ESTIMATE method to determine whether there were any variations. Overall, 16 immune cell types and 13 immune-associated regulatory mechanisms were included in the 29 immunological markers measured and compared by single sample gene set enrichment analysis (ssGSEA) in the two risk groups using the “GSVA” R package^22^. Finally, we constructed an immune-related heatmap. In timer 2.0 (http://timer.cistrome.org/, until October 29th, 2022), the proportion of immune cells was determined using seven different methods: TIMER, CIBERSORT, CIBERSORT-ABS, QUANTISEQ, XCELL, EPIC, and MCPCOUNTER. Additionally, scatter plots were generated using the CIBERSORT method (P < 0.05) to display the interactions between immune cells and the risk score^23^.

### Somatic cell mutation analysis

Simple nucleotide variation (SNP) data were downloaded from TCGA for LUAD. We utilized the “maftools” package to determine the mutation status of individuals and to estimate the tumor mutation burden (TMB) score for every individual. We then compared the TMB between the two risk groups^24^.

### Clinical pharmacotherapy

Tumor immune dysfunction and exclusion (TIDE) (http://tide.dfci.harvard.edu/, until October 29th, 2022) is a method for simulating immunological escape of tumors and predicts sensitivity to PD1 drugs and CTLA4 drugs, and the probability of immune evasion is positively associated with the TIDE score^25^. We applied the “oncoPredict” R package to predict the susceptibility of LUAD patients to 198 chemotherapeutic agents and to search for the most effective chemotherapeutic drugs for the two risk groups. We defined drugs with P < 0.05 as sensitive drugs^26^.

### Real-time quantitative polymerase chain reaction (RT-qPCR) for human lung adenocarcinoma

At the Second Affiliated Hospital of Nanchang University, we collected 18 lung adenocarcinoma tissues from patients after surgical resection. The samples were quickly frozen and kept in liquid nitrogen at − 80 °C. Informed consent was obtained from all participants, and the study was authorized by the ethics committee of the Second Affiliated Hospital of Nanchang University.

All samples were divided into a high-risk group and a low-risk group. According to the manufacturer’s recommendations, RNA was obtained from LUAD tissues using TRlzol reagent (Life Technologies CA, USA) and then randomly assigned for RT-qPCR analysis. This was followed by reverse transcription using the SureScript First-Strand cDNA Synthesis kit (GeneCopropol, Guangzhou, China) at 45 °C for 1 h. The Applied Biosystems 7500 Fast Real-Time PCR System and BlazeTaq SYBR Green qPCR Master Mix (GeneCopropol, Guangzhou, China) were used to complete the RT-qPCR analysis (Applied Biosystems). To determine the level of RNA expression in each sample, we employed 2^ΔΔCt^ values.

In the Human Protein Atlas (HPA) (https://www.proteinatlas.org/, until January 20th, 2023), we analyzed the variations in protein expression of selected ICD-related genes in normal tissues and LUAD tissues, which provides some evidence for the role of ICDs in LUAD.

### Ethics approval and consent

The current study investigated the publicly available data, and was also approved by the ethics committee of the Second Affiliated Hospital of Nanchang University (Nanchang, China). All methods were carried out in accordance with the Declaration of Helsinki.

### Informed consent

Written informed consent was obtained from all participants included in the study.

## Results

### Identification of ICD-related lncRNAs significantly associated with prognosis

**Figure 2:**
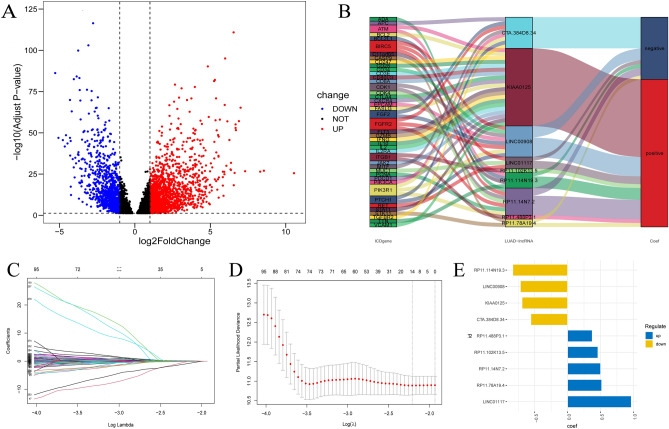
Screening of ICD-related lncRNAs for model construction. (**A**) The volcano plot of differential analysis; (**B**) Correspondence between IRG and the nine ICD-related lncRNAs; (**C**,**D**) 14 ICD-related lncRNAs were identified by lasso regression; (**E**) Correlation of nine ICD-related lncRNAs with prognosis in the construct model.

The 138 selected IRGs and 14,831 lncRNAs were analyzed by Pearson correlation analysis to obtain 9928 ICD-related lncRNAs, after which differential analysis was performed to obtain 2679 differentially expressed lncRNAs (Fig. 2A). A total of 585 samples were included in our study (Table S1), and a total of 477 LUAD samples with survival information were arbitrarily classified into a training set and a test set. (Table 1). The training set was subjected to a univariate Cox analysis with P < 0.05, resulting in 280 ICD-related lncRNAs correlated with prognosis, of which 191 with hazard ratios (HRs) < 1 were protective factors and 89 with HRs > 1 were risk factors (Table S2). After performing LASSO regression analysis, we chose the most appropriate λ value to extract 14 ICD-related lncRNAs (Fig. 2C,D). Finally, we screened a total of 9 noncoexpressed ICD-related lncRNAs (CTA.384D8.34, LINC00908, KIAA0125, LINC01117, RP11.488P3.1, RP11.114N19.3, RP11.14N7.2, RP11.78A19.4 and RP11.102K13.5) using multivariate Cox analysis (Fig. 2E). Specific information on the 9 risk ICD-related lncRNAs is shown in Table S3. We visualized the association of these 9 risk ICD-related lncRNAs with IRGs (Fig. 2B).

**Table 1:** Clinical information of 477 LUAD samples in the TCGA database.

Feature	Train cohort		Test cohort		Entire cohort	
	(n = 239)		(n = 238)		(n = 477)	
	n	%	n	%	n	%
Status						
Alive	151	63.18	151	63.45	302	63.31
Dead	88	36.82	87	36.55	175	36.69
Age						
< = 65	109	45.61	121	50.84	230	48.22
> 65	130	54.39	117	49.16	247	51.78
Gender						
Female	118	49.37	141	59.24	259	54.30
Male	121	50.63	97	40.76	218	45.70
Stage						
Stage I	122	51.05	136	57.14	258	54.09
Stage II	68	28.45	48	20.17	116	24.32
Stage III	37	15.48	41	17.23	78	16.35
Stage IV	12	5.02	13	5.46	25	5.24
T stage						
T1	74	30.96	89	37.39	163	34.17
T2	133	55.65	118	49.58	251	52.62
T3	23	9.62	20	8.40	43	9.01
T4	8	3.35	9	3.78	17	3.56
Unknown	1	0.42	2	0.84	3	0.63
M stage						
M0	157	65.69	159	66.81	316	66.25
M1	11	4.60	13	5.46	24	5.03
Unknown	71	29.71	66	27.73	137	28.72
N stage						
N0	149	62.34	160	67.23	309	64.78
N1	55	23.01	35	14.71	90	18.87
N2	32	13.39	35	14.71	67	14.05
N3	0	0.00	2	0.84	2	0.42
Unknown	3	1.26	6	2.52	9	1.89

*LUAD* lung adenocarcinoma, *TCGA* The Cancer Genome Atlas, *T* tumor, *N* node, *M* metastasis.

### Development of a prognostic model and internal validation

We constructed a prognostic model using 9 filtered ICD-related lncRNAs, and risk scores were determined for every sample in the three cohorts. Risk score = Exp (CTA.384D8.34) × (− 0.557615273) + Exp (LINC00908) × (− 0.716805923) + Exp (KIAA0125) × (− 0.692196258) + Exp (LINC01117) × (0.967632641) + Exp (RP11.488P3.1) × (0.374158171) + Exp (RP11.114N19.3) × (− 0.831794158) + Exp (RP11.14N7.2) × (0.501522434) + Exp (RP11.78A19.4) × (0.517470278) + Exp (RP11.102K13.5) × (0.460623178). The classification of the high-risk group and low-risk group was performed utilizing the middle value for each cohort, as shown for the entire cohort grouping in Table 2.

**Table 2:** Clinical features of LUAD patients in two risk groups.

Feature	High-risk group		Low-risk group	
	(n = 238)		(n = 239)	
	n	%	n	%
Status				
Alive	124	52.10	178	74.48
Dead	114	47.90	61	25.52
Age				
< = 65	123	51.68	107	44.77
> 65	115	48.32	132	55.23
Gender				
Female	120	50.42	139	58.16
Male	118	49.58	100	41.84
Stage				
Stage I	108	45.38	150	62.76
Stage II	66	27.73	50	20.92
Stage III	47	19.75	31	12.97
Stage IV	17	7.14	8	3.35
T stage				
T1	70	29.41	93	38.91
T2	130	54.62	121	50.63
T3	26	10.92	17	7.11
T4	9	3.78	8	3.35
Unknown	3	1.26	0	0.00
M stage				
M0	159	66.81	157	65.69
M1	16	6.72	8	3.35
Unknown	63	26.47	74	30.96
N stage				
N0	137	57.56	172	71.97
N1	54	22.69	36	15.06
N2	42	17.65	25	10.46
N3	0	0.00	2	0.84
Unknown	5	2.10	4	1.67

*LUAD* lung adenocarcinoma.

**Figure 3:**
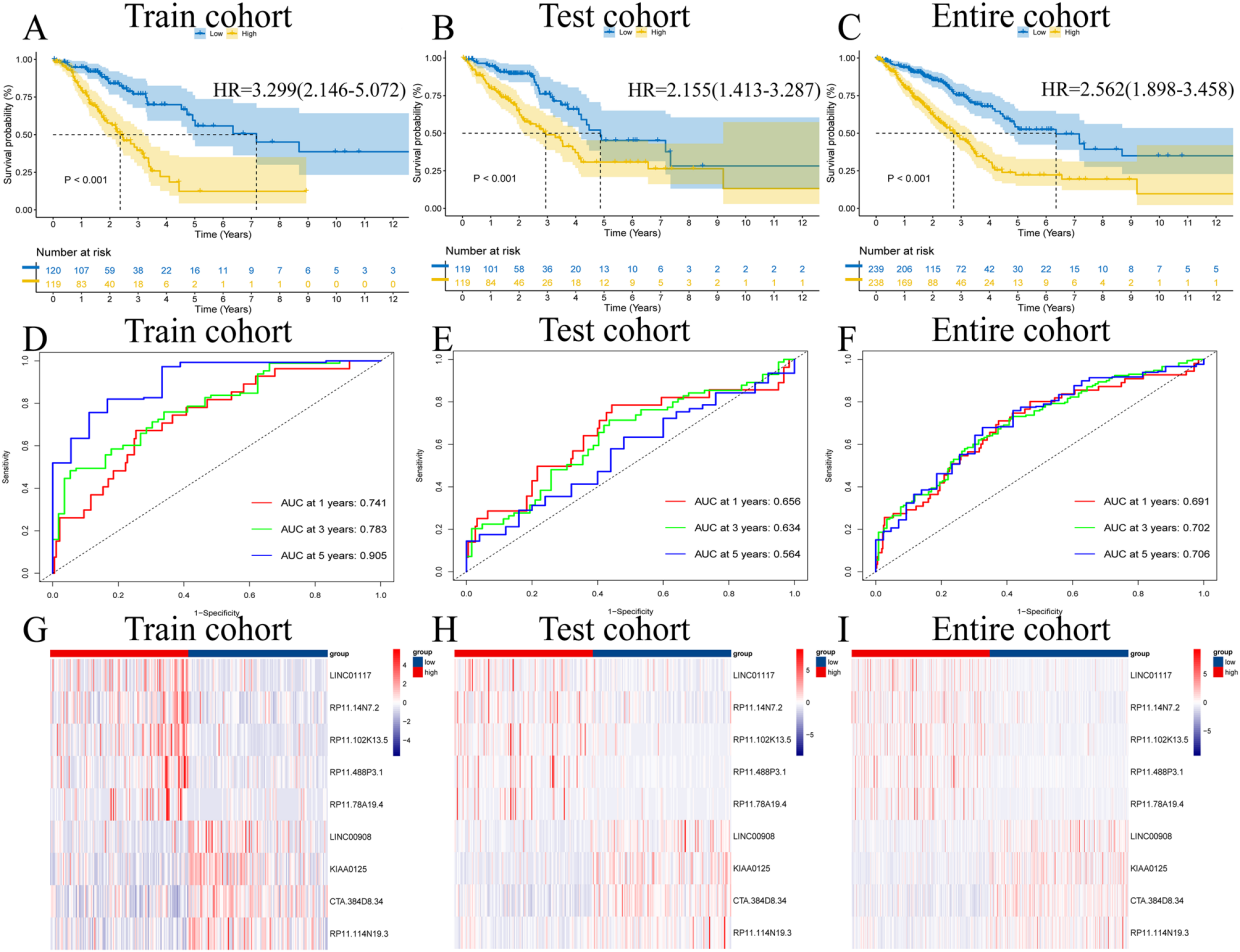
Internal validation of prognostic models. (**A**–**C**) Kaplan–Meier analysis; (**D**–**F**) Time-ROC curves; (**G**–**I**) Heatmap of 9 risk-ICD-related lncRNAs expressions.

According to K‒M analysis, in all three cohorts, the low-risk group had a longer life expectancy than the high-risk group (P < 0.001) (Fig. 3A–C). The reliability of estimating survival rates for 1 year, 3 years, and 5 years was evaluated using the AUC values in time-ROC curves (training: 0.741, 0.783, 0.905; testing: 0.656, 0.634, 0.564; entire: 0.691, 0.702, 0.706) (Fig. 3D–F). Heatmap illustrating the expression of nine ICD-related lncRNAs modeled among the three cohorts (Fig. 3G–I). People with the disease in the low-risk group had a better chance of surviving than those in the high-risk group, according to risk plots for the three cohorts (Fig. S1A–F). PCA was performed with all genes, all lncRNAs, all ICD-related lncRNAs, and risk ICD-related lncRNAs for model construction, in which only risk ICD-related lncRNAs could separate the two risk groups better (Fig. S1G–J). A clinical correlation heatmap revealed that the expression landscape of the nine risk ICD-related lncRNAs modeled was not related to other clinical features (Fig. S2). In addition, K‒M analysis showed that the constructed model was fit to predict the prognosis for all LUAD patients (age ≤ 65, age > 65; female, male; stage I–II, stage III–IV; T1–T2, T3–T4; N0, N1–3; M0, M1) (Fig. S3).

### Constructing a nomogram and anticipating patient survival

**Figure 4:**
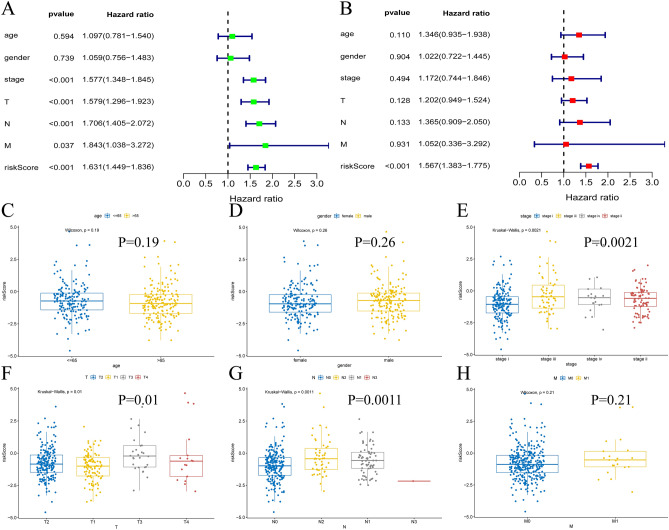
Verify that risk score is an independent predictive indicator. (**A**) Univariate cox regression analysis; (**B**) Multivariate cox regression analysis; (**C**–**H**) Correlation of different clinical characteristics with risk score, including age, gender, stage, T, N, M.

**Figure 5:**
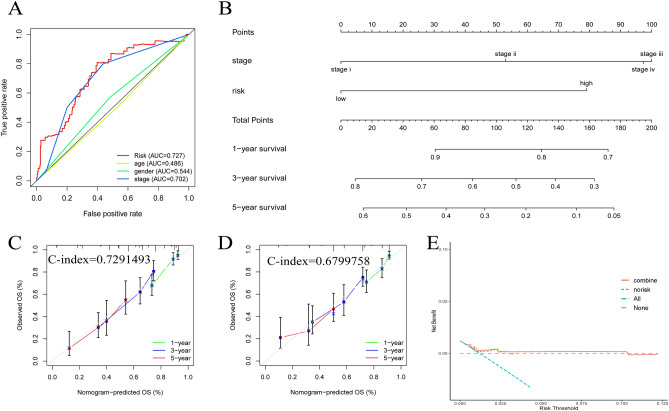
Constructing Nomogram and verifying its feasibility. (**A**) ROC curves based on different clinical factors; (**B**) Construct a nomogram to predict LUAD prognosis; (**C**) Calibration curve for nomogram with stage and risk score; (**D**) Calibration curve for nomogram without risk score; (**E**) Decision clinical curve.

Univariate (P < 0.001, HR = 1.631) and multivariate Cox analyses (P < 0.001, HR = 1.567) were performed (Fig. 4A,B), and the risk score was shown to be an individual indicator to predict patient prognosis (Table S4). In contrast to age and gender, we found that M stage (P = 0.0021), T stage (P = 0.01), and N stage (P = 0.0011) were considerably linked with the risk score (Fig. 4C–H). Then, we drew ROC curves and obtained AUC values (risk score = 0.727, stage = 0.702, gender = 0.544, age = 0.486), revealing that the risk score showed superior predictive value over other clinical indicators (Fig. 5A). A nomogram was constructed on the basis of stage and risk, and different patients could be scored by this system to allow prediction of rates of survival at 1 year, 3 years, and 5 years (Fig. 5B). Then, we chose a patient for successful validation (Fig. S4). Calibration curves with risk showed that the prediction curve highly overlapped with the best prediction line of 45° (C-index = 0.7291493), which had a better fit than the nomogram without risk (C-index = 0.6799758) (Fig. 5C,D). DCA indicated that the model with the risk score had a better clinical benefit than the model without the risk score (Fig. 5E).

### Functional analysis

**Figure 6:**
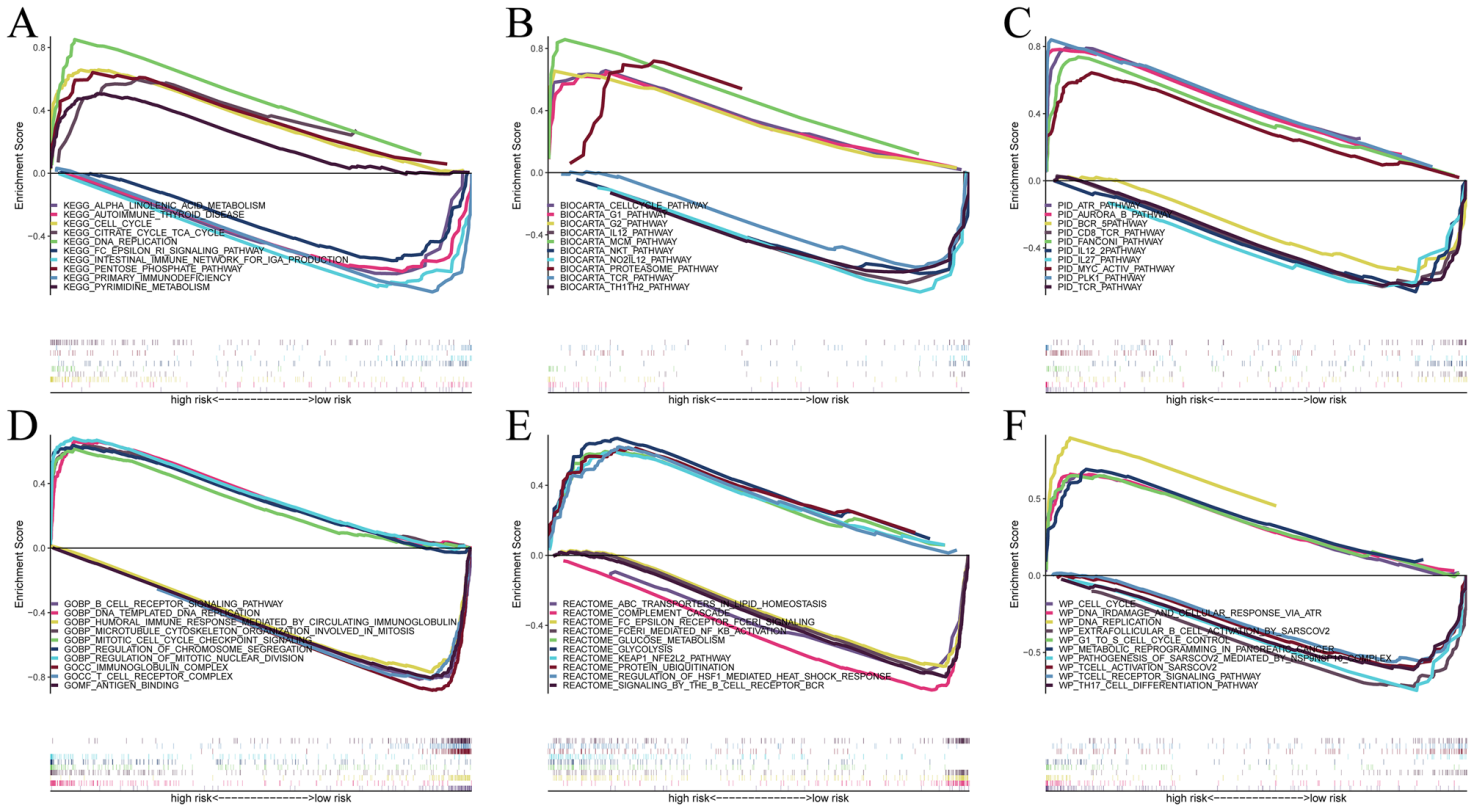
Enrichment analysis: (**A**) KEGG; (**B**) BIOCARTA; (**C**) PID; (**D**) GO; (**E**) REACTOME; (**F**) WIKIPATHWAYS.

We performed enrichment analysis using five different pathway libraries in GSEA, and the results were similar. The high-risk group showed particular upregulation of pathways that are engaged in DNA replication and metabolism. For example, in the KEGG database, cellular pathways such as DNA replication, cell cycle and citrate cycle TCA cycle were significantly enriched in the high-risk group. In the low-risk group, the pathways that were engaged in immunity and inflammation were particularly upregulated. For example, in the WP database, cellular pathways such as the T-cell receptor signaling pathway, T-cell activation sarscov2 and Th17 cell differentiation pathway were significantly enriched in the low-risk group (Fig. 6A–F).

### Tumor immune microenvironment analysis

**Figure 7:**
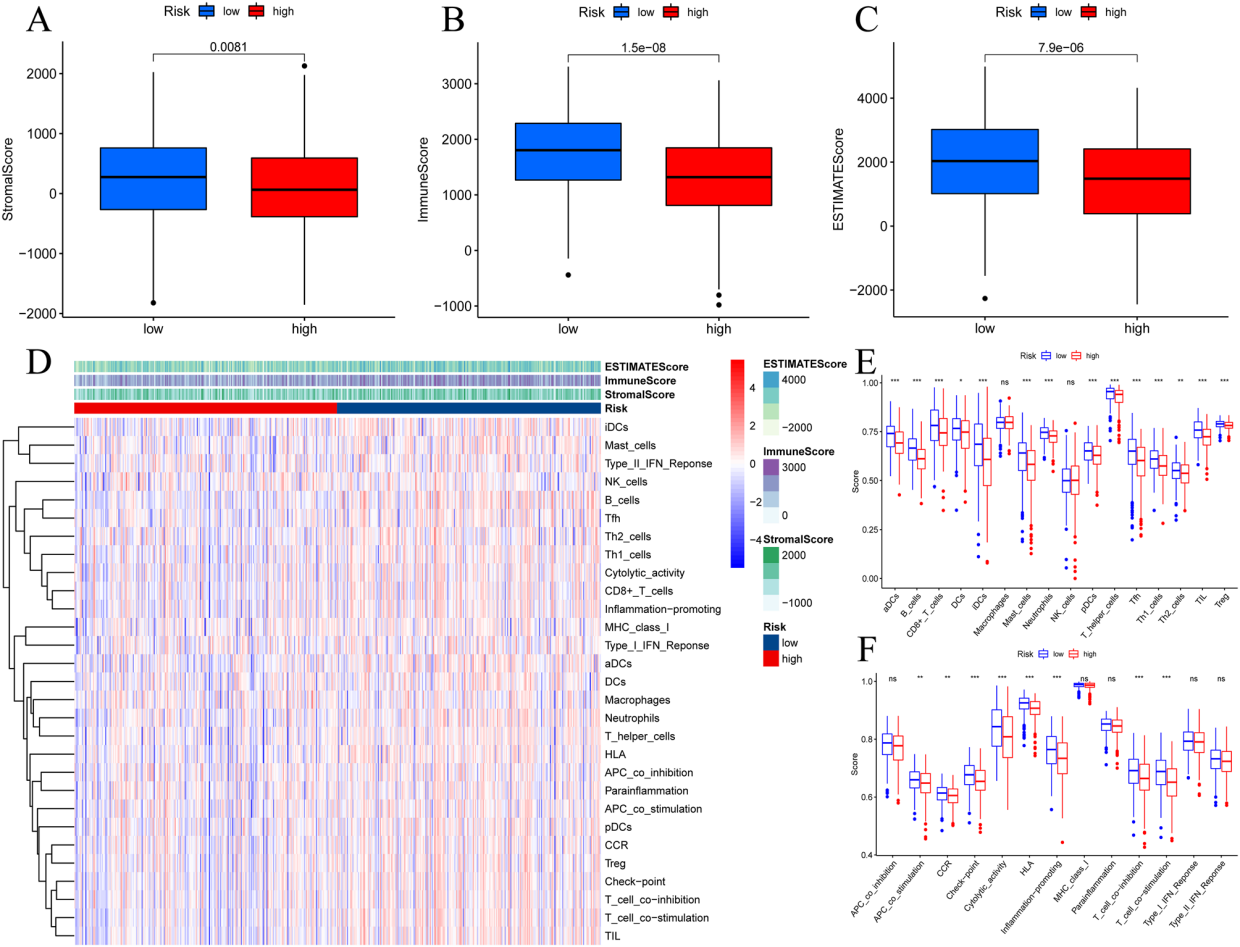
Tumor immune microenvironment analysis. (**A**–**C**) Comparing the differences in stromal score, immune score, and estimate score between two risk groups; (**D**) Differential infiltration of tumor immune microenvironment in two risk groups; (**E**,**F**) Boxplot displaying the expression of 16 immune cells and 13 immune function pathways in two risk groups based on ssGSEA algorithm.

High-risk group members presented a relatively lower average stromal score (P = 0.0081), a lower immune score (P < 0.001), and a lower ESTIMATE score (P < 0.001) than low-risk group members (Fig. 7A–C). The low-risk group presented a state of moderate activity and had more immune cells and immune functional pathways (Fig. 7E,F). Tumor purity is the proportion of tumor cells to all cells in the sample. A lower estimate score indicates a lower proportion of stromal and immune cells in the tumor and, conversely, a higher proportion of tumor cells, and in the study, we can conclude that the high-risk group suffered from high tumor purity and low immune-related marker expression (Fig. 7D). Higher concentrations of most immune cells corresponded to lower risk scores based on seven different immune algorithms, in which the CIBERSORT algorithm resulted in P < 0.05 immune cells (Fig. S5A). The majority of eosinophils, activated memory CD4 T cells, M0 macrophages and M2 macrophages had a higher proportion in the high-risk group. The majority of memory B cells, plasma cells, resting memory CD4 T cells and regulatory Tregs had a higher proportion in the low-risk group (Fig. S5B–I).

### Somatic mutation analysis

**Figure 8:**
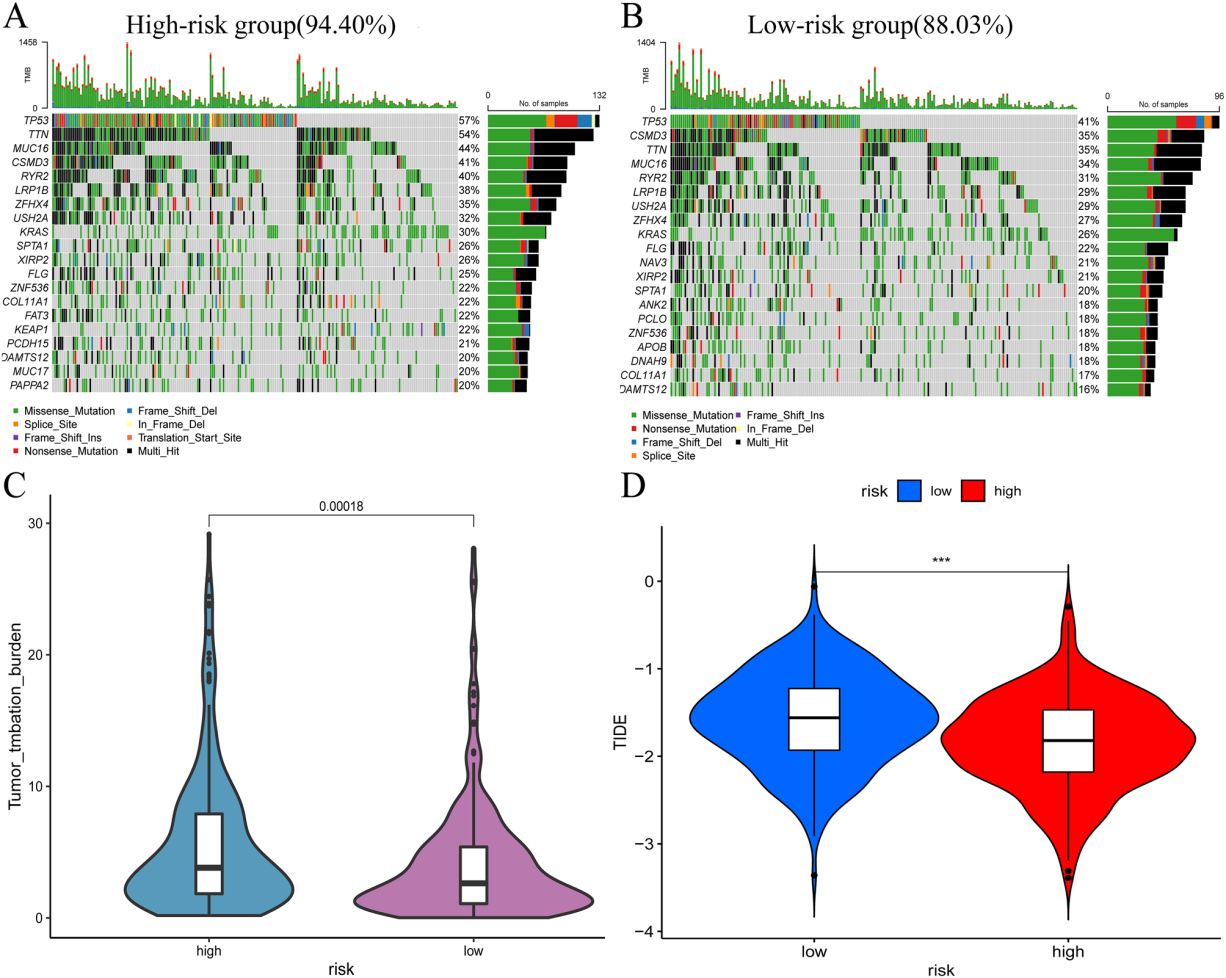
Somatic mutation analysis and immunotherapy. (**A**,**B**) Top 20 genes with the highest mutation frequency in tumor cells of the two risk groups; (**C**) Comparison of TMB between two risk groups; (**D**) Comparison of TIDE scores between two risk groups.

We mapped the first 20 genes with the most frequent mutations in the two risk groups, and the most commonly altered gene in both groups was TP53 (Fig. 8A,B). We found that the top 5 genes with the highest mutation rates in the two risk groups were TP53, TTN, MUC16, CSMD3, and RYR2. Mutations in these five genes may be closely related to LUAD progression. In comparison to the low-risk group, the TMB was substantially higher in the high-risk group (P = 0.00018), indicating that these patients may experience a worse result and respond more favorably to immunotherapy (Fig. 8C).

### Immunotherapy and chemotherapy

The TIDE results showed notably lower scores in the high-risk group than in the low-risk group (P < 0.001), suggesting that the high-risk group was more amenable to immunological treatment (Fig. 8D). Eleven anticancer medicines were effective in the high-risk group, primarily by blocking the EGFR and ERK-MAPK signaling pathways (Table S5). The low-risk group was susceptible to 116 anticancer agents, mainly by inhibiting the PI3K/mTOR signaling pathway and DNA replication (Table S6). In both groups, there were no differences in sensitivity to 68 chemotherapeutic agents (Table S7).

### Biological validation

The HPA database was used to compare the protein expression of ICD-related genes in normal tissues and LUAD tissues by cellular immunohistochemical staining. The protein expression of the genes is shown in Fig. S6A. The primer sequences of nine ICD-related lncRNAs modeled for RT-qPCR are shown in Table S8. The RT-qPCR results showed that the nine ICD-related lncRNAs used to construct the model were differentially expressed in the two risk groups, which was consistent with our study (Fig. S6B).

## Discussion

One of the principal reasons for human mortality is cancer^27^. More than 350 deaths are caused by LC daily. LUAD is the main type of LC, and its incidence remains high^1^. Traditional TNM staging still has significant limitations for assessing patient prognosis and selecting treatment options^4^. New predictive tools need to be discovered^28^. Selective induction of cancer cell death is the most effective way to fight cancer^29^. When tumor cells are stimulated externally, the conversion of nonimmunogenicity to immunogenicity mediates the induction of ICD by the body to generate an antitumor immune response^8^. The high-risk group showed active pathways that are associated with DNA replication and metabolism. The immune system of patients in the high-risk group remained suppressed. The comparatively high TMB and low TIDE scores in the high-risk group suggest that these patients might be more susceptible to anti-PD1 and anti-CTLA4 treatments. The two risk groups had dissimilar susceptibilities to distinct chemotherapeutic agents.

The construction of prognostic models using ICD-related lncRNAs and the prediction of cancer patient prognosis have been employed for a variety of tumor types, including HGGs, HNSC and GC^11–13^. Our findings were consistent with these previous studies, with the high-risk group experiencing a poorer outcome and the nomogram used to assess patient prognosis having a better predictive value, suggesting that prognostic features constructed based on ICD-related lncRNAs are a good predictive tool for assessing patient prognosis. Therefore, to construct a similar prognostic model, we identified nine ICD-related lncRNAs via Pearson analysis, univariate Cox analysis, LASSO regression, and multivariate Cox analysis. To accurately forecast the survival rate, we created a nomogram that was successfully validated. Our model has great scientific validity, and two ICD-related lncRNAs affecting tumor mechanisms were investigated. Signal transducer and activator of transcription is negatively regulated when LINC00908 is knocked down, encouraging the growth of tumors^30^. Based on our constructed model, the high-risk group had low LINC00908 expression and a worse prognosis, consistent with previous findings. KIAA0125 overexpression has also been shown to inhibit tumor cell proliferation, metastasis and infiltration through Wnt/β-linked protein signaling^31^, which is consistent with our study, where high KIAA0125 expression in the low-risk group inhibited tumor proliferation and metastasis, leading to a better prognosis. Subsequently, we explored the molecular mechanisms involved in the progression of tumors by enrichment analysis, and to understand the efficacy of immunotherapy in LUAD patients, we performed tumor microenvironmental analysis, tumor mutation burden, and TIDE. Finally, we predicted the sensitivity of LUAD patients to various chemotherapeutic agents, which provides a basis for the clinical search for appropriate drugs.

GSEA was conducted in the two risk groups to identify potential pathways, and immunological and inflammatory-associated pathways were notably enriched in the low-risk group, which indicated a close correlation between the development of LUAD and immunity. A large percentage of neoplastic cells may be killed when ICD is activated in the low-risk group, improving prognosis^8^. Pathways related to metabolism and DNA replication were more abundant in the high-risk group, among which the pentose phosphate pathway (PPP) not only synthesizes pentose phosphate to provide nucleic acids as a raw material for cancer cell proliferation but also generates nicotinamide adenine dinucleotide phosphate (NADPH), which is necessary for redox reactions in the metabolism of various substances. Activation of the PPP allows cancer cells to metabolize nutrients and actively proliferate, promoting tumor progression. Some tumor suppressors, such as TP53, are also associated with PPP regulation, and in this study, we astonishingly observed that TP53 showed the highest mutation level in the high-risk group. Moreover, TP53 deletion has been shown to reduce the ability of TP53 to prevent cancer cells from absorbing glucose, contributing to overactivation of the PPP and resulting in a poor outcome^32^. The high-risk group is immunosuppressed, and the immunosuppressive state of most tumors can significantly limit ICD-driven immunity to clear tumor cells. In addition, poor immune cell infiltration, including antigen-presenting cells and their precursor cells, means that dead tumor cells are less likely to be effectively processed and drive ICD, which leads to a poor prognosis^8^.

A higher TMB indicates a higher possibility of neoantigens and a higher immune reaction rate^33^. In the high-risk group, the TMB was higher and the TIDE scores were lower. According to these findings, the high-risk group seems to be more responsive to immunological therapies, including anti-PD1 and anti-CTLA4 agents. In contrast, the TIDE scores remained higher in the low-risk group, indicating a higher likelihood of immunological escape. Patients with early-stage lung cancer rely mainly on surgical resection, and in patients with advanced lung adenocarcinoma, EGFR inhibitors usually have good benefits^34^. In our study, the high-risk group was found to be sensitive to EGFR inhibitors, such as erlotinib, lapatinib, and gefitinib, which are already being used as the principal treatments for LC.

We present the following innovative ideas in our study. First, we are the first to develop a prognostic model using ICD-related lncRNAs to predict the outcome of patients with LUAD. Second, our risk model has a better prediction effect than alternative clinical indicators. Moreover, we compared a nomogram constructed using only the traditional stage to a nomogram that included the risk model and found that the latter had a better prediction capacity. Third, we found that Lu et al. developed a pattern of necroptosis-related lncRNAs to predict overall survival in LUAD^35^. We performed somatic mutation analysis, immunotherapy analysis, and drug sensitivity analysis, which they did not perform. Furthermore, the AUC value of their constructed model (0.723) was smaller than that of ours (0.727). Therefore, our model is more useful in assessing the prognosis of LUAD patients and selecting treatments. Although our results have good scientific validity, there are still shortcomings. First, we only chose the TCGA database for the analysis, without external validation. Second, the specific mechanisms of action of the ICD-related lncRNAs in LUAD investigated in our study are still not clear and need to be explored in subsequent experiments. The advancement of interaction prediction research in various fields of computational biology would provide valuable insights into genetic markers and related diseases, such as the NDALMA model and GCNCRF model for predicting interactions between lncRNAs and miRNAs^36,37^, the GFPA model and the scAAGA model for processing single-cell data^38,39^, the MDA-AENMF model and the GCNAT model for forecasting potential relationships between metabolites and disease^40,41^, and the DMFGAM model for developing related drugs^42^. In follow-up studies, we can make predictions by developing or using similar models to investigate the mechanism of action of lncRNAs in LUAD and the clinical development of related drugs. Third, this thesis only considered the role of ICD in LUAD, but there are many other types of cell death within the cell. Similar to what Li et al. found, whether our screened lncRNAs also bind to different substances (e.g., miRNAs, proteins) to drive droplet assembly and mediate different types of cell death modes needs to be explored^43,44^. This may help us to have a more in-depth understanding of disease mechanisms and therapeutic targets.

## Conclusion

Based on 9 ICD-related lncRNAs, we developed a predictive model and a nomogram that has great value in assessing prognosis and directing clinical therapy in LUAD patients. The presence of DNA replication and metabolism-related pathways and an immunosuppressed state in the high-risk group likely results in a poorer outcome. The risk model is also valuable in guiding the choice of antineoplastic agents for LUAD patients. The RT-qPCR results also further confirmed the accuracy of this model. Due to the above shortcomings, this study still requires basic research to further confirm the specific mechanisms, and the selection of clinically relevant drugs must be verified through clinical practice.

### Supplementary Information

Supplementary Information.

## Data Availability

The data sets used and/or analyzed during the current study are available from the corresponding author upon reasonable request.

## Abbreviations

AUC: The area under curves
Coef: Coefficient
DCA: Decision curve analysis
FDR: False discovery rate
GO: Gene ontology
GSEA: Gene set enrichment analysis
H: High
HPA: The Human Protein Atlas
HR: Hazard ratios
ICD: Immunogenic cell death
ICD-related-lncRNA: Immunogenic-cell-death related long non-coding RNA
IRG: Immunogenic-cell-death related gene
KEGG: Kyoto Encyclopedia of Genes and Genomes
K–M survival analysis: Kaplan–Meier survival analysis
L: Low
LC: Lung cancer
LncRNA: Long non-coding RNA
LUAD: Lung adenocarcinoma carcinoma
NES: Normalized Enrichment Score
P: Probability
PCA: Principal component analysis
PID: Pathway Interaction Database
RT-qPCR: Real-time quantitative polymerase chain reaction
ROC analysis: Time receiver-operating characteristic analysis
RS: Risk score
Se (coef): Standard error (coefficient)
SNP: Simple nucleotide variation
ssGSEA: Single sample gene set enrichment analysis
TCGA: The Cancer Genome Atlas
TIDE: Tumor immune dysfunction and exclusion
TMB: Tumor mutation burden
TNM: Tumor node metastasis
Z: Z scores (standard deviation)

## References

[1] Siegel RL, Miller KD, Fuchs HE, Jemal A (2022). Cancer statistics, 2022. CA Cancer J. Clin..

[2] Xu JY (2020). Integrative proteomic characterization of human lung adenocarcinoma. Cell.

[3] Ettinger DS (2022). Non-small cell lung cancer, version 3.2022, NCCN clinical practice guidelines in oncology. J. Natl. Compr. Cancer Netw..

[4] Thai AA, Solomon BJ, Sequist LV, Gainor JF, Heist RS (2021). Lung cancer. Lancet.

[5] Liu Z (2022). Machine learning-based integration develops an immune-derived lncRNA signature for improving outcomes in colorectal cancer. Nat. Commun..

[6] Liang YL (2022). A lncRNA signature associated with tumor immune heterogeneity predicts distant metastasis in locoregionally advanced nasopharyngeal carcinoma. Nat. Commun..

[7] Buttner FA (2022). A novel molecular signature identifies mixed subtypes in renal cell carcinoma with poor prognosis and independent response to immunotherapy. Genome Med..

[8] Galluzzi L (2020). Consensus guidelines for the definition, detection and interpretation of immunogenic cell death. J. Immunother. Cancer.

[9] Liu T (2022). Cancer-associated fibroblast-specific lncRNA LINC01614 enhances glutamine uptake in lung adenocarcinoma. J. Hematol. Oncol..

[10] Zhang H (2022). Machine learning-based tumor-infiltrating immune cell-associated lncRNAs for predicting prognosis and immunotherapy response in patients with glioblastoma. Brief Bioinform..

[11] Ding D (2022). Prognostic value of antitumor drug targets prediction using integrated bioinformatic analysis for immunogenic cell death-related lncRNA model based on stomach adenocarcinoma characteristics and tumor immune microenvironment. Front. Pharmacol..

[12] Tang X (2022). Upregulated immunogenic cell-death-associated gene signature predicts reduced responsiveness to immune-checkpoint-blockade therapy and poor prognosis in high-grade gliomas. Cells.

[13] Wang X (2021). An immunogenic cell death-related classification predicts prognosis and response to immunotherapy in head and neck squamous cell carcinoma. Front. Immunol..

[14] Ritchie ME (2015). limma powers differential expression analyses for RNA-sequencing and microarray studies. Nucleic Acids Res..

[15] Wazni OM (2021). Cryoballoon ablation as initial therapy for atrial fibrillation. N. Engl. J. Med..

[16] Mandrekar JN (2010). Receiver operating characteristic curve in diagnostic test assessment. J. Thorac. Oncol..

[17] Duforet-Frebourg N, Luu K, Laval G, Bazin E, Blum MG (2016). Detecting genomic signatures of natural selection with principal component analysis: Application to the 1000 genomes data. Mol. Biol. Evol..

[18] Vrieze SI (2012). Model selection and psychological theory: A discussion of the differences between the Akaike information criterion (AIC) and the Bayesian information criterion (BIC). Psychol. Methods.

[19] Gafita A (2021). Nomograms to predict outcomes after (177)Lu-PSMA therapy in men with metastatic castration-resistant prostate cancer: An international, multicentre, retrospective study. Lancet Oncol..

[20] Van Calster B (2018). Reporting and interpreting decision curve analysis: A guide for investigators. Eur. Urol..

[21] Ma X, Yang S, Jiang H, Wang Y, Xiang Z (2021). Transcriptomic analysis of tumor tissues and organoids reveals the crucial genes regulating the proliferation of lung adenocarcinoma. J. Transl. Med..

[22] Wang F (2022). CDC6 is a prognostic biomarker and correlated with immune infiltrates in glioma. Mol. Cancer.

[23] Newman AM (2015). Robust enumeration of cell subsets from tissue expression profiles. Nat. Methods.

[24] Mayakonda A, Lin DC, Assenov Y, Plass C, Koeffler HP (2018). Maftools: Efficient and comprehensive analysis of somatic variants in cancer. Genome Res..

[25] Jiang P (2018). Signatures of T cell dysfunction and exclusion predict cancer immunotherapy response. Nat. Med..

[26] Maeser D, Gruener RF, Huang RS (2021). oncoPredict: An R package for predicting in vivo or cancer patient drug response and biomarkers from cell line screening data. Brief. Bioinform..

[27] Liu S (2022). Serum exosomal proteomics analysis of lung adenocarcinoma to discover new tumor markers. BMC Cancer.

[28] Kim N, Jeong D, Jo A, Eum HH, Lee HO (2022). Prescreening of tumor samples for tumor-centric transcriptome analyses of lung adenocarcinoma. BMC Cancer.

[29] Leng L (2014). Molecular imaging for assessment of mesenchymal stem cells mediated breast cancer therapy. Biomaterials.

[30] Wang Y (2020). LncRNA-encoded polypeptide ASRPS inhibits triple-negative breast cancer angiogenesis. J. Exp. Med..

[31] Hong JY (2021). A catenin of the plakophilin-subfamily, Pkp3, responds to canonical-Wnt pathway components and signals. Biochem. Biophys. Res. Commun..

[32] Patra KC, Hay N (2014). The pentose phosphate pathway and cancer. Trends Biochem. Sci..

[33] Bravaccini S, Bronte G, Ulivi P (2021). TMB in NSCLC: A broken dream?. Int. J. Mol. Sci..

[34] Duma N, Santana-Davila R, Molina JR (2019). Non-small cell lung cancer: Epidemiology, screening, diagnosis, and treatment. Mayo Clin. Proc..

[35] Lu Y (2022). A Novel necroptosis-related lncRNA signature predicts the prognosis of lung adenocarcinoma. Front. Genet..

[36] Zhang L, Yang P, Feng H, Zhao Q, Liu H (2021). Using network distance analysis to predict lncRNA–miRNA interactions. Interdiscip. Sci..

[37] Wang W, Zhang L, Sun J, Zhao Q, Shuai J (2022). Predicting the potential human lncRNA–miRNA interactions based on graph convolution network with conditional random field. Brief. Bioinform..

[38] Hu H (2023). Gene function and cell surface protein association analysis based on single-cell multiomics data. Comput. Biol. Med..

[39] Meng R, Yin S, Sun J, Hu H, Zhao Q (2023). scAAGA: Single cell data analysis framework using asymmetric autoencoder with gene attention. Comput. Biol. Med..

[40] Gao H (2023). Predicting metabolite-disease associations based on auto-encoder and non-negative matrix factorization. Brief. Bioinform..

[41] Sun F, Sun J, Zhao Q (2022). A deep learning method for predicting metabolite-disease associations via graph neural network. Brief. Bioinform..

[42] Wang T, Sun J, Zhao Q (2023). Investigating cardiotoxicity related with hERG channel blockers using molecular fingerprints and graph attention mechanism. Comput. Biol. Med..

[43] Li X (2022). Caspase-1 and gasdermin D afford the optimal targets with distinct switching strategies in NLRP1b inflammasome-induced cell death. Research (Wash D C).

[44] Xu F (2023). Specificity and competition of mRNAs dominate droplet pattern in protein phase separation. Phys. Rev. Res..

