# Supplementary material for: Prognostic value and immune landscapes of immunogenic cell death-associated lncRNAs in lung adenocarcinoma
Source: Sci Rep. 2023 Nov 6;13:19151. doi: 10.1038/s41598-023-46669-w (PMC10628222; doi:10.1038/s41598-023-46669-w)
Supplement: Supplementary file 1 — Supplementary Information. [file 41598_2023_46669_MOESM1_ESM.docx]

**Supplementary files**

**Figure S1** Risk plots and PCA. (A, C, E) Risk curve for risk score; (B, D, F) Risk point diagram of the survival status of each patient; (G-J) Principal component analysis (PCA), including all gene, all lncRNAs, ICD-related lncRNAs, ICD-related lncRNAs for model.


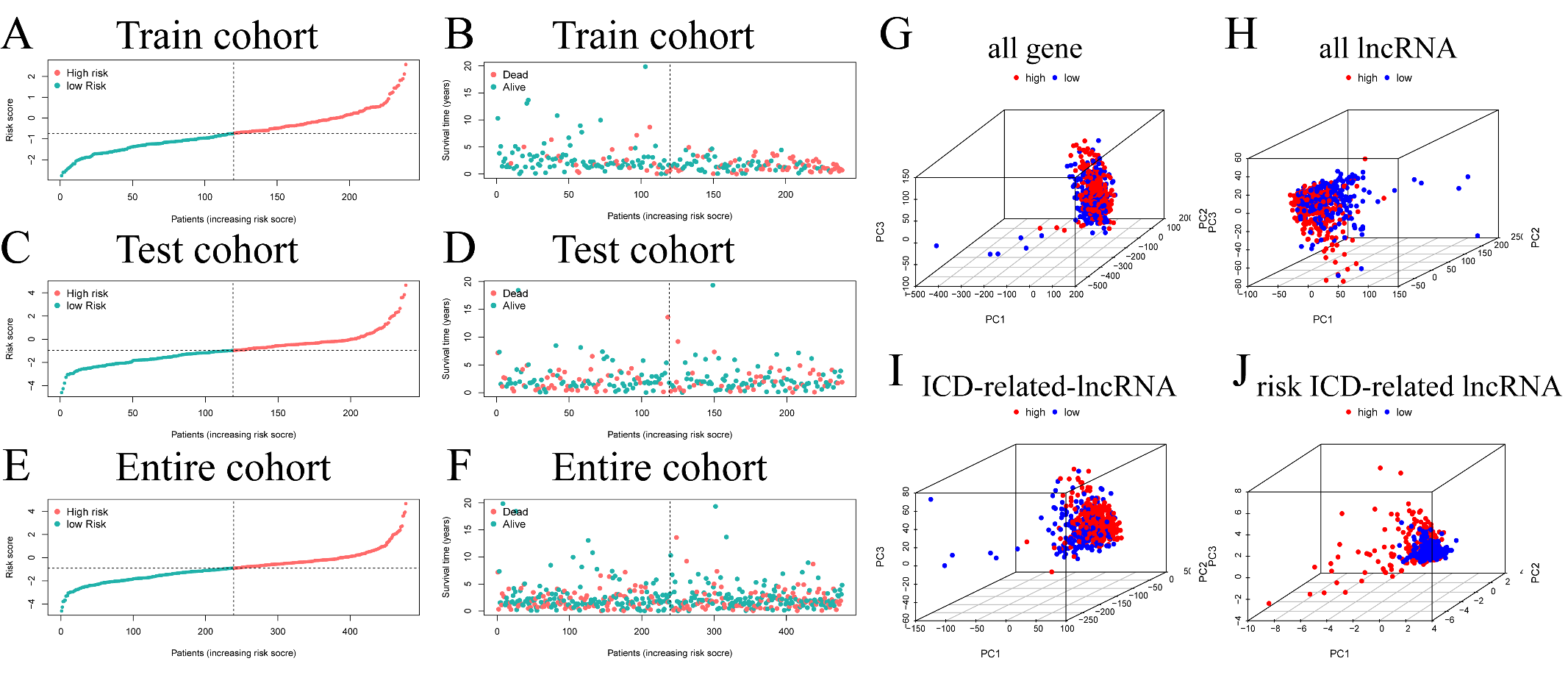


**Figure S2** Clinical relevance heatmap.


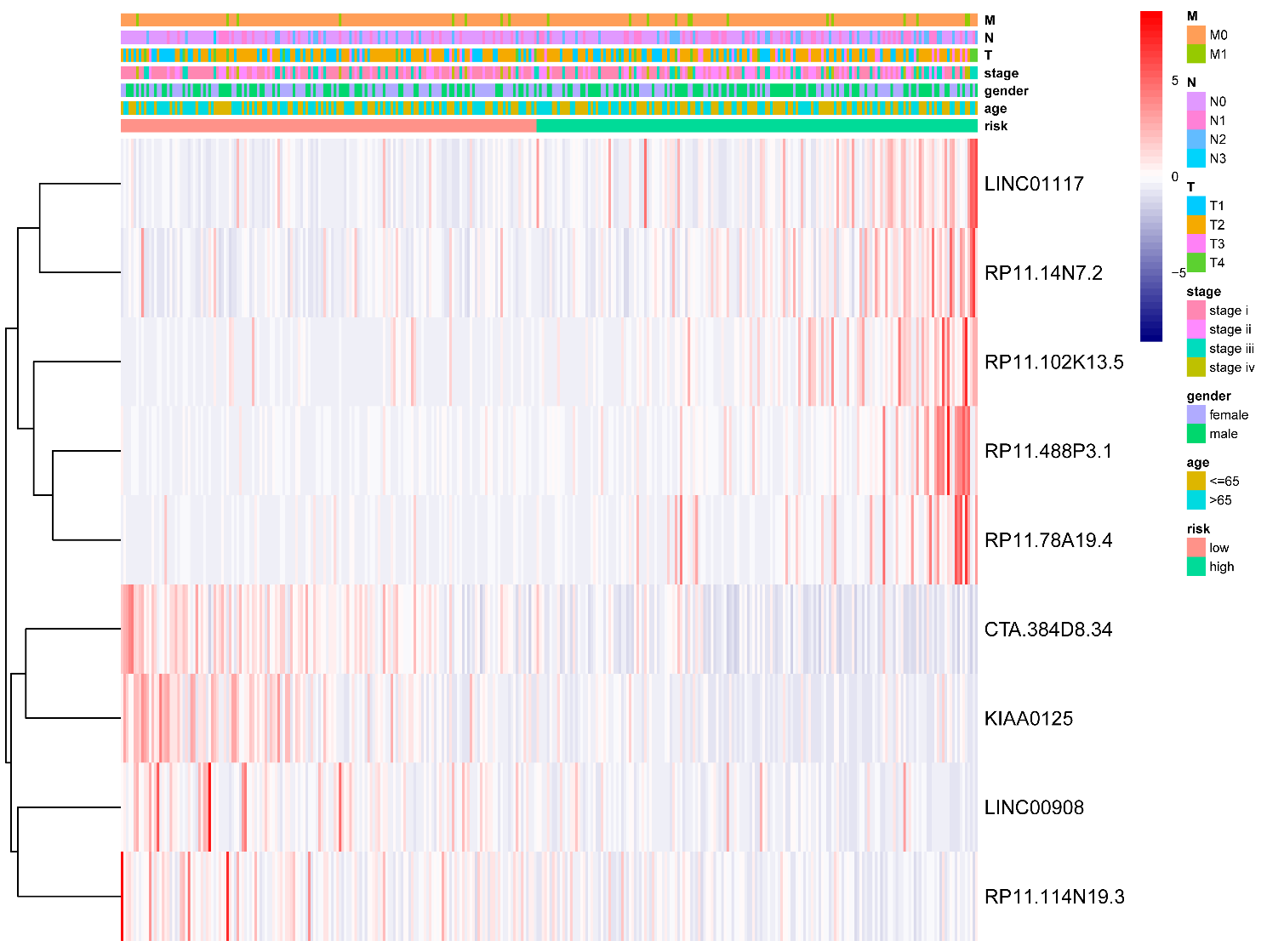


**Figure S3** Survival analysis of a different population: (A) age<=65; (B) age>65; (C) female; (D) male; (E) stage Ⅰ-Ⅱ; (F) stage Ⅲ-Ⅳ; (G) T1-T2; (H) T3-T4; (I) N0; (J) N1-N3; (K) M0; (L) M1.


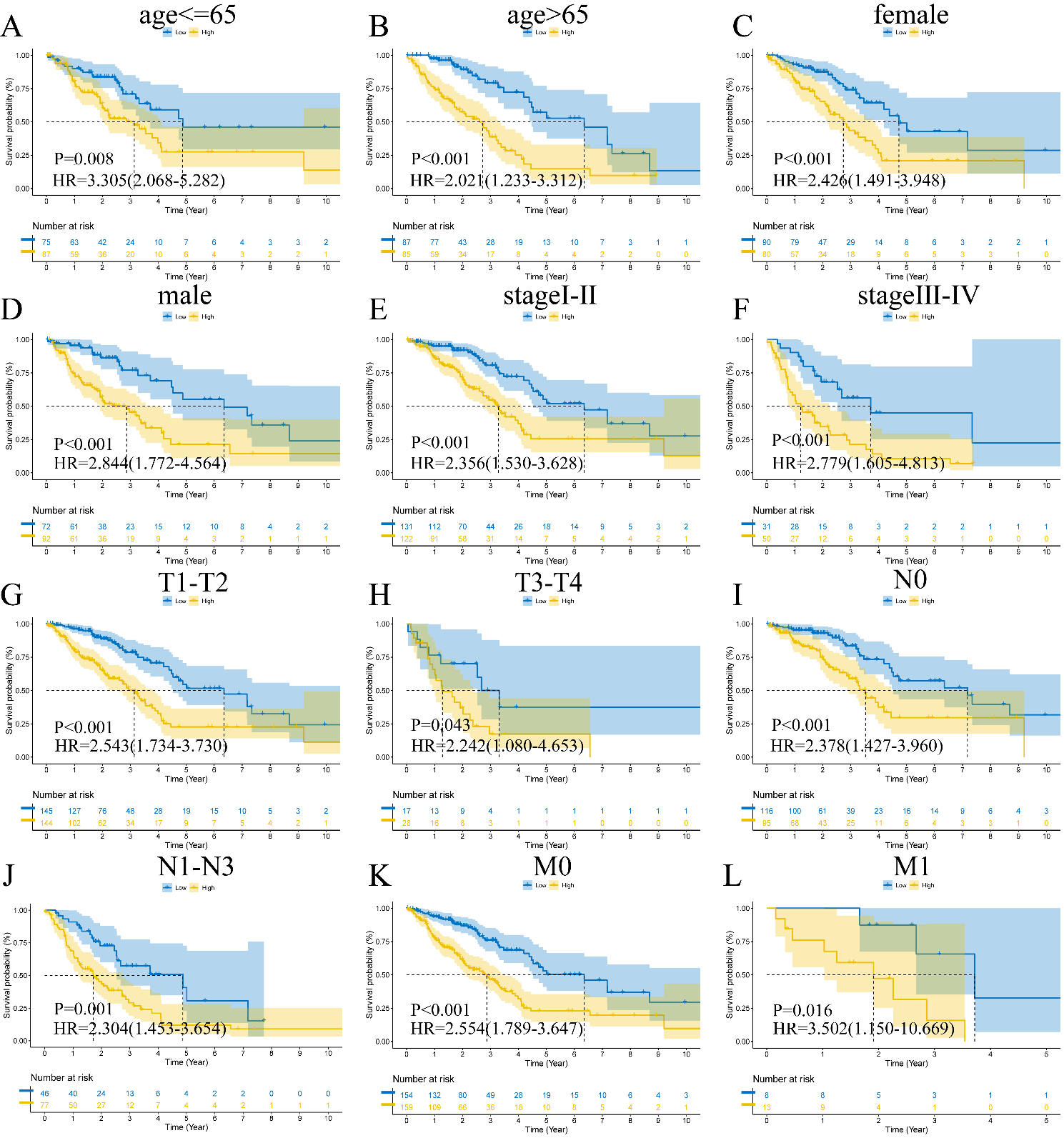


**Figure S4** Validating the nomogram's ability to anticipate.


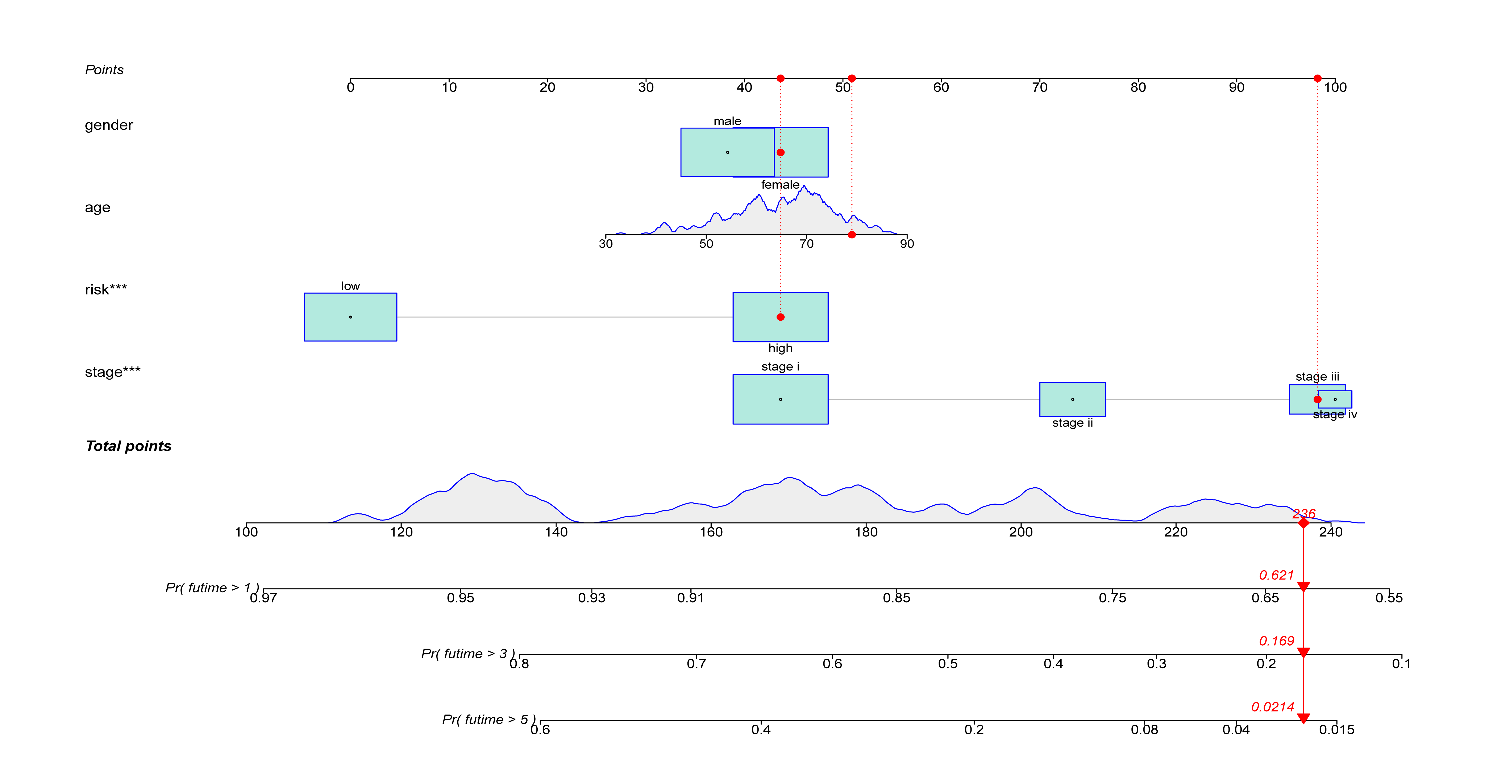


**Figure S5** Correlation of immune cell concentrations and risk scores. (A) Bubble plots of the correlation between different immune cells and risk scores based on seven immune algorithms; (B-I) Scatter plot of the correlation between immune cells and risk scores based on the CIBERSORT algorithm P<0.05.


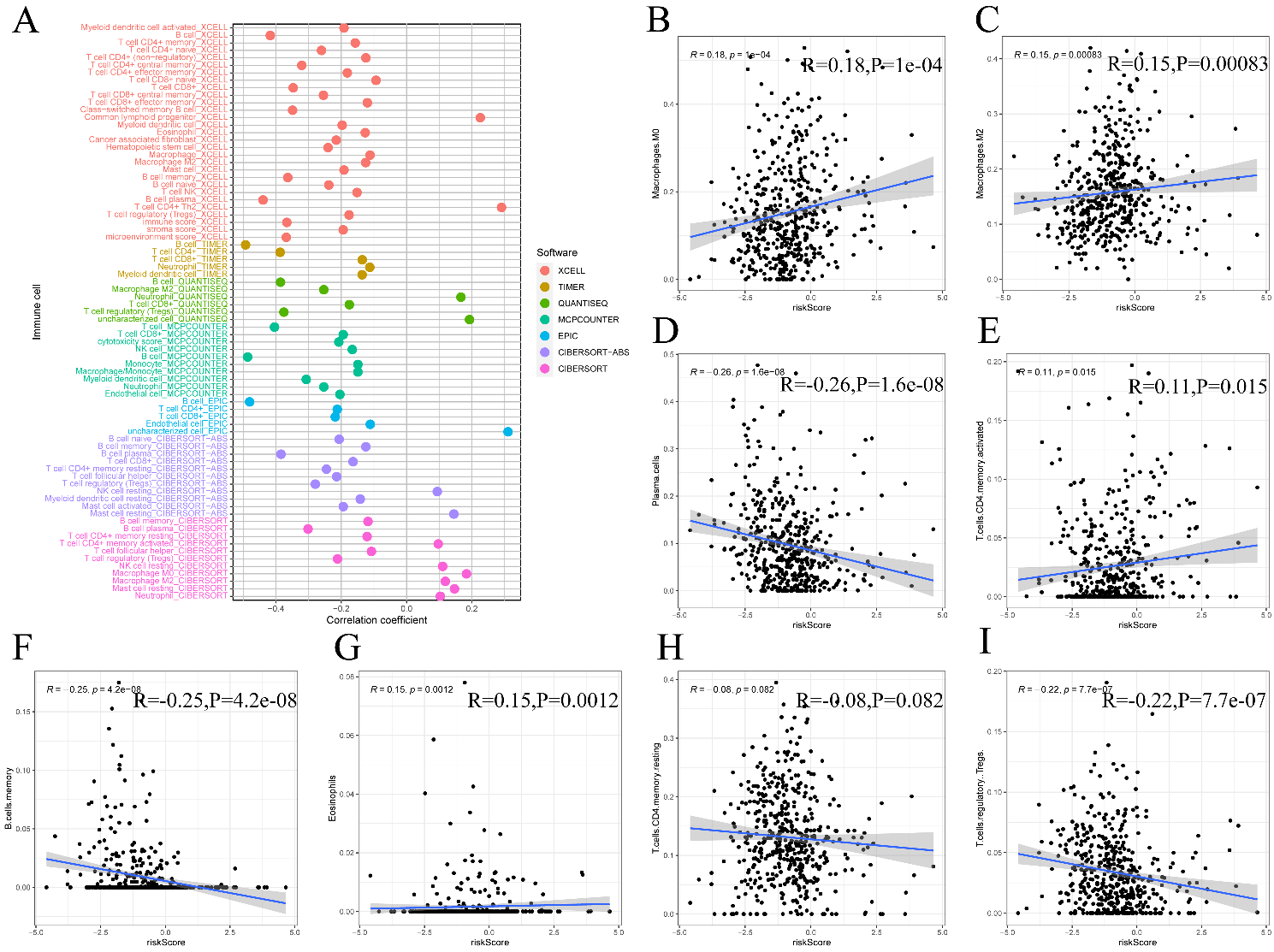


**Figure S6** Biological validation. (A) Compare the protein expression of ICD-related genes between normal tissues and LUAD tissues based on immunohistochemical staining of HPA; (B) The result of RT-qPCR shows the expression of nine ICD-related lncRNAs.


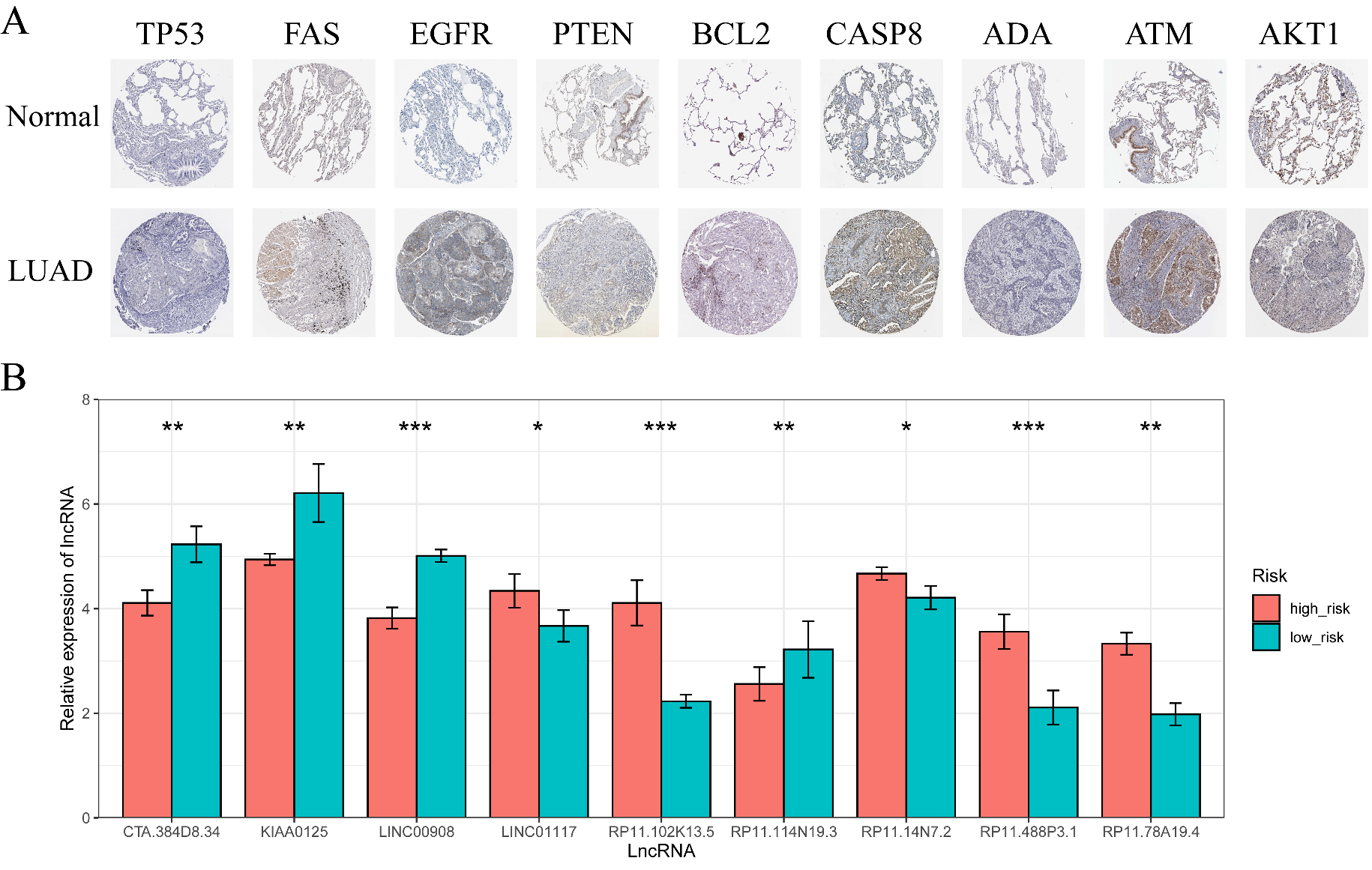


**TableS1** Summary of 585 sample IDs downloaded from the TCGA database.

| **Sample ID** | **Sample ID** | **Sample ID** |
| --- | --- | --- |
| TCGA.97.7938.01A | TCGA.73.4676.11A | TCGA.86.A456.01A |
| TCGA.55.7574.01A | TCGA.55.8505.01A | TCGA.91.7771.01A |
| TCGA.05.4250.01A | TCGA.99.8028.01A | TCGA.67.4679.01B |
| TCGA.55.6979.11A | TCGA.38.6178.01A | TCGA.50.8457.01A |
| TCGA.95.A4VK.01A | TCGA.55.6987.01A | TCGA.80.5608.01A |
| TCGA.97.A4M6.01A | TCGA.83.5908.01A | TCGA.86.6851.01A |
| TCGA.78.7155.01A | TCGA.55.6543.01A | TCGA.05.4384.01A |
| TCGA.05.4402.01A | TCGA.64.1678.01A | TCGA.64.1681.01A |
| TCGA.91.6831.11A | TCGA.91.6835.01A | TCGA.44.7671.01A |
| TCGA.MP.A4T4.01A | TCGA.S2.AA1A.01A | TCGA.55.8511.01A |
| TCGA.78.7535.01A | TCGA.69.7761.01A | TCGA.MP.A4TC.01A |
| TCGA.55.8619.01A | TCGA.86.8672.01A | TCGA.75.7025.01A |
| TCGA.95.7567.01A | TCGA.44.5644.01A | TCGA.55.8302.01A |
| TCGA.49.6745.01A | TCGA.MP.A4SW.01A | TCGA.91.6835.11A |
| TCGA.44.6777.11A | TCGA.MP.A4SV.01A | TCGA.44.2657.11A |
| TCGA.MP.A4TI.01A | TCGA.44.6779.01A | TCGA.NJ.A4YF.01A |
| TCGA.44.3396.11A | TCGA.97.A4LX.01A | TCGA.55.6970.01A |
| TCGA.50.6673.01A | TCGA.99.8025.01A | TCGA.55.8620.01A |
| TCGA.55.1592.01A | TCGA.55.A57B.01A | TCGA.50.5941.01A |
| TCGA.44.8117.01A | TCGA.MN.A4N1.01A | TCGA.78.7539.01A |
| TCGA.50.6594.01A | TCGA.38.4628.01A | TCGA.L9.A743.01A |
| TCGA.62.8402.01A | TCGA.35.4123.01A | TCGA.50.5939.11A |
| TCGA.50.5045.01A | TCGA.50.5930.01A | TCGA.73.A9RS.01A |
| TCGA.J2.A4AD.01A | TCGA.73.4677.01A | TCGA.44.2665.01A |
| TCGA.4B.A93V.01A | TCGA.69.7764.01A | TCGA.L9.A8F4.01A |
| TCGA.67.3770.01A | TCGA.49.4501.01A | TCGA.55.7725.01A |
| TCGA.05.5715.01A | TCGA.44.3918.01A | TCGA.MP.A4TE.01A |
| TCGA.50.8460.01A | TCGA.44.2655.01A | TCGA.86.8673.01A |
| TCGA.05.4249.01A | TCGA.05.4434.01A | TCGA.49.6744.11A |
| TCGA.86.A4P8.01A | TCGA.44.6145.01A | TCGA.86.8074.01A |
| TCGA.49.6742.11A | TCGA.38.4625.11A | TCGA.86.A4D0.01A |
| TCGA.55.A494.01A | TCGA.78.7150.01A | TCGA.MP.A4T8.01A |
| TCGA.50.5931.11A | TCGA.38.4626.01A | TCGA.44.5643.01A |
| TCGA.95.8494.01A | TCGA.91.6840.01A | TCGA.97.8552.01A |
| TCGA.55.7726.01A | TCGA.J2.A4AE.01A | TCGA.69.A59K.01A |
| TCGA.44.6147.11A | TCGA.50.5932.01A | TCGA.86.7701.01A |
| TCGA.78.8660.01A | TCGA.62.A46R.01A | TCGA.62.A470.01A |
| TCGA.49.4490.01A | TCGA.05.4405.01A | TCGA.55.7727.01A |
| TCGA.05.4410.01A | TCGA.55.6986.11A | TCGA.62.8398.01A |
| TCGA.44.2659.01A | TCGA.75.6212.01A | TCGA.78.7145.01A |
| TCGA.78.7633.01A | TCGA.97.7554.01A | TCGA.67.6215.01A |
| TCGA.55.8299.01A | TCGA.73.4675.01A | TCGA.55.7907.01A |
| TCGA.67.6217.01A | TCGA.MN.A4N5.01A | TCGA.MP.A4T9.01A |
| TCGA.67.3774.01A | TCGA.91.6828.01A | TCGA.86.8669.01A |
| TCGA.MP.A4T6.01A | TCGA.93.8067.01A | TCGA.49.AARR.01A |
| TCGA.50.5942.01A | TCGA.97.8176.01A | TCGA.97.7941.01A |
| TCGA.55.6969.11A | TCGA.55.A493.01A | TCGA.44.A47B.01A |
| TCGA.86.8280.01A | TCGA.62.A46S.01A | TCGA.55.7227.01A |
| TCGA.49.6761.11A | TCGA.55.6983.11A | TCGA.55.8097.01A |
| TCGA.44.7662.01A | TCGA.55.6968.01A | TCGA.97.8171.01A |
| TCGA.78.7146.01A | TCGA.44.6147.01A | TCGA.99.7458.01A |
| TCGA.97.8179.01A | TCGA.55.7911.01A | TCGA.55.8089.01A |
| TCGA.55.7570.01A | TCGA.73.4658.01A | TCGA.05.4424.01A |
| TCGA.50.5072.01A | TCGA.44.6778.01A | TCGA.62.8395.01A |
| TCGA.49.AAQV.01A | TCGA.75.6205.01A | TCGA.55.A490.01A |
| TCGA.86.8671.01A | TCGA.75.6203.01A | TCGA.64.1676.01A |
| TCGA.91.6829.01A | TCGA.L9.A444.01A | TCGA.50.5936.01A |
| TCGA.78.7537.01A | TCGA.64.5779.01A | TCGA.55.A48Y.01A |
| TCGA.91.8497.01A | TCGA.44.7660.01A | TCGA.93.7348.01A |
| TCGA.69.7973.01A | TCGA.44.2666.01A | TCGA.38.4630.01A |
| TCGA.NJ.A55A.01A | TCGA.49.4512.11A | TCGA.05.5423.01A |
| TCGA.91.6849.01A | TCGA.78.8640.01A | TCGA.55.8621.01A |
| TCGA.49.AAR9.01A | TCGA.44.6777.01A | TCGA.05.4397.01A |
| TCGA.97.7552.01A | TCGA.44.2661.11A | TCGA.55.7910.01A |
| TCGA.55.8092.01A | TCGA.J2.8194.01A | TCGA.J2.A4AG.01A |
| TCGA.75.6214.01A | TCGA.55.7903.01A | TCGA.86.8073.01A |
| TCGA.49.6744.01A | TCGA.97.8174.01A | TCGA.44.4112.01B |
| TCGA.64.1680.01A | TCGA.38.4627.11A | TCGA.44.2666.01B |
| TCGA.95.7043.01A | TCGA.99.8033.01A | TCGA.75.5146.01A |
| TCGA.55.8091.01A | TCGA.55.1594.01A | TCGA.NJ.A4YQ.01A |
| TCGA.44.5645.11A | TCGA.55.6980.01A | TCGA.55.7995.01A |
| TCGA.73.4676.01A | TCGA.MP.A4TK.01A | TCGA.44.7659.01A |
| TCGA.55.6982.01A | TCGA.55.8207.01A | TCGA.55.1596.01A |
| TCGA.62.A46Y.01A | TCGA.44.A4SS.01A | TCGA.69.7979.01A |
| TCGA.44.2656.01A | TCGA.50.5933.01A | TCGA.55.7724.01A |
| TCGA.L4.A4E5.01A | TCGA.44.7672.01A | TCGA.55.6972.01A |
| TCGA.50.5051.01A | TCGA.44.6146.01B | TCGA.44.3919.01A |
| TCGA.49.AARQ.01A | TCGA.67.3771.01A | TCGA.J2.8192.01A |
| TCGA.78.7161.01A | TCGA.67.3772.01A | TCGA.55.6968.11A |
| TCGA.05.4426.01A | TCGA.97.A4M5.01A | TCGA.44.A47A.01A |
| TCGA.97.A4M0.01A | TCGA.53.A4EZ.01A | TCGA.38.4629.01A |
| TCGA.44.8120.01A | TCGA.99.8032.01A | TCGA.86.A4JF.01A |
| TCGA.78.7147.01A | TCGA.73.4670.01A | TCGA.69.8453.01A |
| TCGA.91.6836.11A | TCGA.55.7576.01A | TCGA.55.6985.11A |
| TCGA.86.7713.01A | TCGA.49.6743.11A | TCGA.91.8496.01A |
| TCGA.67.3773.01A | TCGA.05.4425.01A | TCGA.50.6595.11A |
| TCGA.44.A479.01A | TCGA.93.A4JN.01A | TCGA.95.7039.01A |
| TCGA.05.4422.01A | TCGA.55.6983.01A | TCGA.NJ.A4YP.01A |
| TCGA.49.4494.01A | TCGA.75.7027.01A | TCGA.62.A46O.01A |
| TCGA.91.6830.01A | TCGA.62.A472.01A | TCGA.44.6147.01B |
| TCGA.44.3398.11B | TCGA.78.7154.01A | TCGA.73.4668.01A |
| TCGA.44.7669.01A | TCGA.50.5044.01A | TCGA.05.4433.01A |
| TCGA.50.5066.01A | TCGA.95.A4VP.01A | TCGA.95.A4VN.01A |
| TCGA.64.5815.01A | TCGA.86.8674.01A | TCGA.78.7156.01A |
| TCGA.50.5939.01A | TCGA.97.A4M1.01A | TCGA.38.A44F.01A |
| TCGA.MP.A4TA.01A | TCGA.49.AARO.01A | TCGA.05.4396.01A |
| TCGA.64.5778.01A | TCGA.91.6831.01A | TCGA.55.8204.01A |
| TCGA.44.5645.01B | TCGA.55.6642.01A | TCGA.55.7815.01A |
| TCGA.80.5611.01A | TCGA.86.8056.01A | TCGA.55.7281.01A |
| TCGA.44.2662.01A | TCGA.L9.A5IP.01A | TCGA.78.7166.01A |
| TCGA.05.5429.01A | TCGA.97.7546.01A | TCGA.55.6982.11A |
| TCGA.38.4632.01A | TCGA.97.7547.01A | TCGA.55.8614.01A |
| TCGA.O1.A52J.01A | TCGA.44.6776.11A | TCGA.44.6148.11A |
| TCGA.75.6206.01A | TCGA.NJ.A55O.01A | TCGA.78.7152.01A |
| TCGA.50.5946.02A | TCGA.55.7914.01A | TCGA.64.5781.01A |
| TCGA.64.5774.01A | TCGA.78.7143.01A | TCGA.86.8075.01A |
| TCGA.05.4418.01A | TCGA.50.5936.11A | TCGA.MP.A4TD.01A |
| TCGA.64.1677.01A | TCGA.55.8205.01A | TCGA.86.8281.01A |
| TCGA.55.8507.01A | TCGA.05.4415.01A | TCGA.55.8513.01A |
| TCGA.55.6984.11A | TCGA.86.8358.01A | TCGA.78.7542.01A |
| TCGA.05.5425.01A | TCGA.91.6847.01A | TCGA.49.4487.01A |
| TCGA.49.4510.01A | TCGA.86.8585.01A | TCGA.44.2662.11A |
| TCGA.78.8655.01A | TCGA.55.8203.01A | TCGA.05.4390.01A |
| TCGA.55.6979.01A | TCGA.71.6725.01A | TCGA.86.8668.01A |
| TCGA.NJ.A7XG.01A | TCGA.55.7816.01A | TCGA.73.4659.01A |
| TCGA.91.8499.01A | TCGA.55.6978.01A | TCGA.35.5375.01A |
| TCGA.50.7109.01A | TCGA.44.6775.01A | TCGA.91.6829.11A |
| TCGA.97.8547.01A | TCGA.44.2662.01B | TCGA.86.6562.01A |
| TCGA.49.4514.01A | TCGA.49.4506.01A | TCGA.86.8279.01A |
| TCGA.78.7153.01A | TCGA.86.7954.01A | TCGA.49.6767.01A |
| TCGA.55.6971.01A | TCGA.78.8662.01A | TCGA.44.6776.01A |
| TCGA.44.3918.01B | TCGA.MP.A4TF.01A | TCGA.55.6981.01A |
| TCGA.49.AARE.01A | TCGA.MP.A4T7.01A | TCGA.95.7944.01A |
| TCGA.69.8254.01A | TCGA.44.5645.01A | TCGA.55.8615.01A |
| TCGA.49.6761.01A | TCGA.55.8506.01A | TCGA.97.7553.01A |
| TCGA.69.7980.01A | TCGA.50.6591.01A | TCGA.05.4403.01A |
| TCGA.69.8253.01A | TCGA.L4.A4E6.01A | TCGA.55.8512.01A |
| TCGA.95.7947.01A | TCGA.49.4505.01A | TCGA.78.7536.01A |
| TCGA.49.4490.11A | TCGA.44.4112.01A | TCGA.55.6981.11A |
| TCGA.95.8039.01A | TCGA.67.6216.01A | TCGA.91.6848.01A |
| TCGA.62.8394.01A | TCGA.55.8508.01A | TCGA.53.7626.01A |
| TCGA.55.8616.01A | TCGA.55.8085.01A | TCGA.93.7347.01A |
| TCGA.55.6986.01A | TCGA.62.A471.01A | TCGA.55.6978.11A |
| TCGA.49.6743.01A | TCGA.44.A4SU.01A | TCGA.05.4427.01A |
| TCGA.49.AAR4.01A | TCGA.44.3917.01B | TCGA.44.2657.01A |
| TCGA.35.3615.01A | TCGA.73.7498.01A | TCGA.55.7728.01A |
| TCGA.99.AA5R.01A | TCGA.55.8206.01A | TCGA.50.5049.01A |
| TCGA.05.4417.01A | TCGA.75.6207.01A | TCGA.86.A4P7.01A |
| TCGA.53.7624.01A | TCGA.95.7948.01A | TCGA.78.7162.01A |
| TCGA.97.A4M2.01A | TCGA.91.6828.11A | TCGA.69.8255.01A |
| TCGA.97.A4M3.01A | TCGA.50.5944.01A | TCGA.05.4432.01A |
| TCGA.44.2668.01A | TCGA.50.8459.01A | TCGA.55.A492.01A |
| TCGA.49.AAR0.01A | TCGA.44.6146.11A | TCGA.05.4244.01A |
| TCGA.78.7158.01A | TCGA.49.6742.01A | TCGA.44.2668.11A |
| TCGA.49.4512.01A | TCGA.64.1679.01A | TCGA.50.6597.01A |
| TCGA.86.7711.01A | TCGA.L9.A443.01A | TCGA.MP.A5C7.01A |
| TCGA.44.2656.01B | TCGA.97.A4M7.01A | TCGA.50.6590.01A |
| TCGA.75.5147.01A | TCGA.MP.A4TH.01A | TCGA.78.7160.01A |
| TCGA.44.6775.01C | TCGA.44.6144.11A | TCGA.55.8514.01A |
| TCGA.L9.A50W.01A | TCGA.75.5122.01A | TCGA.49.AAR2.01A |
| TCGA.49.6745.11A | TCGA.50.5930.11A | TCGA.35.4122.01A |
| TCGA.75.5125.01A | TCGA.50.5066.02A | TCGA.93.A4JO.01A |
| TCGA.80.5607.01A | TCGA.55.8087.01A | TCGA.55.8096.01A |
| TCGA.55.8094.01A | TCGA.50.5932.11A | TCGA.55.A48X.01A |
| TCGA.05.4430.01A | TCGA.62.A46P.01A | TCGA.91.6836.01A |
| TCGA.91.A4BD.01A | TCGA.38.4626.11A | TCGA.78.7540.01A |
| TCGA.78.7149.01A | TCGA.50.5935.11A | TCGA.49.4488.01A |
| TCGA.50.6593.01A | TCGA.38.4632.11A | TCGA.78.7148.01A |
| TCGA.86.8054.01A | TCGA.50.6595.01A | TCGA.55.7283.01A |
| TCGA.55.A48Z.01A | TCGA.44.6146.01A | TCGA.44.3917.01A |
| TCGA.05.5420.01A | TCGA.55.7913.01B | TCGA.L9.A7SV.01A |
| TCGA.55.8090.01A | TCGA.49.AAR3.01A | TCGA.55.8208.01A |
| TCGA.44.8119.01A | TCGA.91.6847.11A | TCGA.MN.A4N4.01A |
| TCGA.55.6971.11A | TCGA.55.6985.01A | TCGA.55.6712.01A |
| TCGA.55.7994.01A | TCGA.55.6975.11A | TCGA.97.8172.01A |
| TCGA.69.7763.01A | TCGA.38.4627.01A | TCGA.44.2661.01A |
| TCGA.73.7499.01A | TCGA.55.6975.01A | TCGA.55.A491.01A |
| TCGA.55.8301.01A | TCGA.62.A46V.01A | TCGA.MP.A4SY.01A |
| TCGA.NJ.A4YI.01A | TCGA.55.6984.01A | TCGA.05.4395.01A |
| TCGA.55.7573.01A | TCGA.53.7813.01A | TCGA.44.7667.01A |
| TCGA.75.7030.01A | TCGA.38.4631.01A | TCGA.44.A47G.01A |
| TCGA.91.A4BC.01A | TCGA.49.4507.01A | TCGA.55.6972.11A |
| TCGA.78.7220.01A | TCGA.78.7159.01A | TCGA.05.4389.01A |
| TCGA.05.4382.01A | TCGA.55.8510.01A | TCGA.55.6980.11A |
| TCGA.75.7031.01A | TCGA.86.7955.01A | TCGA.95.7562.01A |
| TCGA.86.8278.01A | TCGA.55.A4DG.01A | TCGA.86.7714.01A |
| TCGA.NJ.A55R.01A | TCGA.44.6148.01A | TCGA.55.6970.11A |
| TCGA.97.8175.01A | TCGA.93.A4JP.01A | TCGA.44.6145.11A |
| TCGA.73.4662.01A | TCGA.50.5931.01A | TCGA.86.8076.01A |
| TCGA.44.2655.11A | TCGA.97.8177.01A | TCGA.50.5068.01A |
| TCGA.44.6774.01A | TCGA.50.5933.11A | TCGA.97.7937.01A |
| TCGA.MP.A4TJ.01A | TCGA.50.5946.01A | TCGA.05.4398.01A |
| TCGA.69.7765.01A | TCGA.50.5055.01A | TCGA.50.6592.01A |
| TCGA.64.5775.01A | TCGA.78.8648.01A | TCGA.44.3396.01A |
| TCGA.44.7661.01A | TCGA.44.6778.11A | TCGA.91.6849.11A |
| TCGA.49.4486.01A | TCGA.71.8520.01A | TCGA.69.7760.01A |
| TCGA.75.6211.01A | TCGA.50.5935.01A | TCGA.55.7284.01B |
| TCGA.49.AARN.01A | TCGA.78.7167.01A | TCGA.55.A4DF.01A |
| TCGA.38.7271.01A | TCGA.69.7978.01A | TCGA.62.8397.01A |
| TCGA.62.8399.01A | TCGA.44.7670.01A | TCGA.78.7163.01A |
| TCGA.73.4666.01A | TCGA.44.2668.01B | TCGA.86.8359.01A |
| TCGA.86.7953.01A | TCGA.69.7974.01A | TCGA.05.4420.01A |
| TCGA.75.5126.01A | TCGA.44.3398.01A | TCGA.38.4625.01A |
| TCGA.86.8055.01A | TCGA.NJ.A4YG.01A | TCGA.93.A4JQ.01A |
| TCGA.44.2665.11A | TCGA.05.5428.01A | TCGA.55.5899.01A |

**Abbreviation:** TCGA: The Cancer Genome Atlas; ID: Identity.

**Table S2** The specific information of 280 ICD-related-lncRNAs screened after univariate cox analysis at P<0.05 based on Train cohort.

| **Gene** | **HR** | **Lower** | **Upper** | **Gene** | **HR** | **Lower** | **Upper** |
| --- | --- | --- | --- | --- | --- | --- | --- |
| FAM83A-AS1 | 1.44 | 1.16 | 1.79 | RP11-384O8.1 | 1.52 | 1.08 | 2.15 |
| RP11-251M1.1 | 0.40 | 0.21 | 0.75 | RP11-213H15.1 | 0.40 | 0.21 | 0.76 |
| FENDRR | 0.61 | 0.39 | 0.95 | TARID | 2.50 | 1.20 | 5.21 |
| AP001189.4 | 0.35 | 0.14 | 0.86 | LINC01117 | 2.15 | 1.41 | 3.28 |
| LINC00968 | 0.36 | 0.15 | 0.90 | RP11-789C17.3 | 3.23 | 1.03 | 10.05 |
| AC018647.3 | 0.02 | 0.00 | 0.31 | RP11-488P3.1 | 1.64 | 1.29 | 2.08 |
| RP4-575N6.4 | 0.39 | 0.19 | 0.80 | AC007386.2 | 0.00 | 0.00 | 0.79 |
| RP11-582J16.4 | 0.16 | 0.03 | 0.77 | IFNG-AS1 | 0.22 | 0.07 | 0.67 |
| RP11-805I24.3 | 0.00 | 0.00 | 0.37 | ATP13A4-AS1 | 0.62 | 0.41 | 0.95 |
| PCAT19 | 0.65 | 0.43 | 0.98 | RP11-323H21.3 | 0.64 | 0.41 | 0.99 |
| RP11-389C8.2 | 0.56 | 0.34 | 0.90 | RP11-114N19.3 | 0.18 | 0.06 | 0.56 |
| EP300-AS1 | 0.59 | 0.37 | 0.93 | RP11-8P13.5 | 1.98 | 1.24 | 3.17 |
| CTA-384D8.35 | 0.79 | 0.65 | 0.96 | RP11-470M17.2 | 0.80 | 0.66 | 0.97 |
| RP1-18D14.7 | 0.01 | 0.00 | 0.84 | RP11-14N7.2 | 1.97 | 1.42 | 2.75 |
| LINC00857 | 1.65 | 1.17 | 2.33 | CTC-340I23.2 | 0.17 | 0.03 | 0.96 |
| RP11-539E17.5 | 2.16 | 1.20 | 3.91 | RP11-162J8.3 | 0.59 | 0.36 | 0.95 |
| RP11-455O6.2 | 0.19 | 0.06 | 0.67 | RP11-968A15.8 | 0.35 | 0.12 | 0.99 |
| AP000438.2 | 0.03 | 0.00 | 0.40 | LINC01010 | 0.33 | 0.12 | 0.91 |
| MED4-AS1 | 0.17 | 0.05 | 0.56 | RP11-41O4.2 | 52.73 | 3.31 | 841.04 |
| LINC00987 | 0.46 | 0.22 | 0.94 | AC079354.5 | 3.50 | 1.13 | 10.80 |
| OR7E47P | 0.52 | 0.29 | 0.92 | RP11-428G5.5 | 0.46 | 0.22 | 0.96 |
| RP11-4B16.3 | 0.21 | 0.05 | 0.82 | RP11-20G13.2 | 0.55 | 0.31 | 0.98 |
| RP11-286H15.1 | 0.45 | 0.20 | 0.98 | AP000439.3 | 1.70 | 1.20 | 2.41 |
| LINC00032 | 0.00 | 0.00 | 0.07 | HOXD-AS2 | 1.72 | 1.08 | 2.74 |
| FTO-IT1 | 0.46 | 0.22 | 0.96 | FAM181A-AS1 | 0.25 | 0.06 | 1.00 |
| LINC01352 | 0.24 | 0.07 | 0.79 | SIRPG-AS1 | 0.01 | 0.00 | 0.50 |
| RP11-10A14.5 | 1.31 | 1.10 | 1.55 | RP6-91H8.3 | 3.18 | 1.33 | 7.62 |
| RP11-98D18.9 | 0.58 | 0.34 | 0.97 | RP11-20G13.3 | 0.48 | 0.23 | 0.97 |
| LINC01447 | 0.00 | 0.00 | 0.92 | RP11-624C23.1 | 0.02 | 0.00 | 0.61 |
| RP11-283G6.4 | 0.33 | 0.17 | 0.65 | RP11-431M7.3 | 0.00 | 0.00 | 0.58 |
| RP11-295G20.2 | 1.22 | 1.03 | 1.44 | RP11-78A19.4 | 2.18 | 1.41 | 3.37 |
| LINC00551 | 0.23 | 0.07 | 0.75 | RP5-887A10.1 | 0.54 | 0.35 | 0.84 |
| RP11-875O11.1 | 0.28 | 0.10 | 0.80 | RP11-603J24.21 | 0.33 | 0.13 | 0.85 |
| RP11-327L3.3 | 0.09 | 0.01 | 0.83 | RP11-725P16.2 | 82.59 | 2.52 | 2704.21 |
| CTD-2510F5.4 | 1.24 | 1.01 | 1.51 | RP11-526A4.1 | 24.47 | 1.01 | 594.99 |
| RP11-475O23.2 | 0.19 | 0.04 | 0.93 | CH507-154B10.2 | 94.49 | 2.43 | 3671.31 |
| RP1-301L19.1 | 0.00 | 0.00 | 0.90 | C20orf166-AS1 | 0.00 | 0.00 | 0.56 |
| SLC2A1-AS1 | 2.17 | 1.05 | 4.50 | RP11-1105O14.1 | 1.58 | 1.14 | 2.21 |
| RP11-434I12.3 | 0.75 | 0.58 | 0.96 | CTC-563A5.2 | 0.05 | 0.01 | 0.50 |
| SNHG3 | 0.74 | 0.56 | 0.98 | AC078852.1 | 0.12 | 0.02 | 0.65 |
| CTA-384D8.34 | 0.69 | 0.53 | 0.88 | AL591893.1 | 22.89 | 1.58 | 331.81 |
| RP11-561I11.3 | 0.05 | 0.00 | 0.97 | RP5-1031D4.2 | 0.13 | 0.02 | 0.81 |
| RP11-2N1.3 | 0.01 | 0.00 | 0.81 | RP11-108K3.3 | 5.48 | 1.18 | 25.52 |
| RP11-290F5.1 | 0.58 | 0.39 | 0.86 | ARHGAP26-AS1 | 0.10 | 0.01 | 0.68 |
| LINC00887 | 3.52 | 1.63 | 7.61 | SC22CB-1D7.1 | 2.00 | 1.14 | 3.53 |
| C6orf3 | 0.26 | 0.09 | 0.76 | RP11-80I3.1 | 0.00 | 0.00 | 0.26 |
| C5orf64 | 0.00 | 0.00 | 0.00 | RP11-148O21.4 | 0.27 | 0.10 | 0.74 |
| AC090616.2 | 0.69 | 0.50 | 0.97 | CCDC13-AS1 | 0.07 | 0.01 | 0.68 |
| RP11-800A3.7 | 0.37 | 0.13 | 1.00 | LINC01116 | 1.46 | 1.18 | 1.80 |
| RP1-244F24.1 | 0.55 | 0.30 | 1.00 | LINC00535 | 0.08 | 0.01 | 0.82 |
| PAN3-AS1 | 0.49 | 0.24 | 0.99 | RP5-906C1.1 | 0.39 | 0.18 | 0.86 |
| NAV2-AS2 | 0.12 | 0.02 | 0.61 | RP11-125O18.1 | 0.11 | 0.02 | 0.73 |
| Z83851.4 | 1.45 | 1.00 | 2.11 | RP11-227H15.4 | 3.36 | 1.63 | 6.92 |
| WWC2-AS2 | 3.53 | 1.13 | 11.06 | CTD-2231H16.1 | 0.76 | 0.60 | 0.96 |
| AF131215.2 | 0.60 | 0.38 | 0.97 | AL122127.25 | 0.37 | 0.18 | 0.76 |
| RP11-325L12.6 | 0.44 | 0.19 | 1.00 | RP11-403A21.1 | 0.52 | 0.28 | 0.96 |
| RP11-1008C21.2 | 0.47 | 0.22 | 0.99 | RP11-245D16.4 | 0.38 | 0.19 | 0.75 |
| AC007879.5 | 3.26 | 1.34 | 7.92 | RP11-23P13.6 | 0.38 | 0.16 | 0.89 |
| RP1-27K12.4 | 1.25 | 1.03 | 1.52 | KIAA0125 | 0.43 | 0.26 | 0.71 |
| OGFRP1 | 2.62 | 1.29 | 5.31 | LINC00543 | 1.57 | 1.11 | 2.22 |
| LINC00211 | 0.00 | 0.00 | 0.27 | ABCA9-AS1 | 3.72 | 1.12 | 12.29 |
| LINC00092 | 0.24 | 0.09 | 0.67 | RP11-115J23.1 | 3.85 | 1.33 | 11.11 |
| GS1-600G8.5 | 0.48 | 0.25 | 0.95 | CTD-2595P9.4 | 0.10 | 0.01 | 0.78 |
| RP11-16E12.2 | 0.67 | 0.50 | 0.90 | LINC00639 | 0.07 | 0.01 | 0.72 |
| RP11-462L8.1 | 1.33 | 1.07 | 1.64 | AF131217.1 | 0.00 | 0.00 | 0.04 |
| C20orf197 | 0.67 | 0.48 | 0.94 | TTC3-AS1 | 0.08 | 0.01 | 0.84 |
| CTD-2006K23.1 | 0.00 | 0.00 | 0.94 | RBPMS-AS1 | 0.67 | 0.49 | 0.92 |
| AF131215.9 | 0.56 | 0.32 | 0.98 | RP11-455F5.5 | 0.43 | 0.25 | 0.75 |
| RP11-254F19.2 | 0.12 | 0.02 | 0.86 | PCAT29 | 0.34 | 0.14 | 0.86 |
| AC005264.2 | 0.04 | 0.00 | 0.90 | CASC19 | 1.39 | 1.08 | 1.78 |
| CTD-2555C10.3 | 1.67 | 1.16 | 2.40 | CTD-2562J17.4 | 0.00 | 0.00 | 0.34 |
| RP11-134G8.5 | 0.54 | 0.32 | 0.90 | RP11-46H11.12 | 0.40 | 0.18 | 0.93 |
| LINC00707 | 1.60 | 1.17 | 2.19 | LINC00996 | 0.30 | 0.15 | 0.61 |
| AC006129.1 | 0.26 | 0.10 | 0.69 | RP11-114M1.1 | 0.59 | 0.40 | 0.86 |
| FGF14-AS2 | 0.69 | 0.47 | 0.99 | KCNQ1-AS1 | 4.84 | 1.53 | 15.31 |
| LINC00628 | 5.09 | 1.21 | 21.45 | AC017006.2 | 0.02 | 0.00 | 0.92 |
| AF127936.3 | 0.24 | 0.07 | 0.88 | RP4-785G19.5 | 1.95 | 1.00 | 3.78 |
| RP11-573G6.6 | 0.07 | 0.01 | 0.38 | RP11-731F5.2 | 0.59 | 0.38 | 0.94 |
| RP11-750H9.5 | 0.66 | 0.46 | 0.92 | RP11-1055B8.3 | 0.24 | 0.07 | 0.82 |
| A2M-AS1 | 0.31 | 0.11 | 0.83 | RP11-1149M10.2 | 0.15 | 0.04 | 0.62 |
| FLJ27354 | 0.30 | 0.09 | 0.96 | RP11-588H23.3 | 0.35 | 0.13 | 0.96 |
| AC093609.1 | 0.17 | 0.04 | 0.85 | RP11-479J7.2 | 2.89 | 1.05 | 7.95 |
| RP11-533E19.7 | 0.37 | 0.15 | 0.90 | RP11-192P3.4 | 0.00 | 0.00 | 0.44 |
| AC007386.4 | 0.40 | 0.22 | 0.74 | RP3-329E20.2 | 0.12 | 0.01 | 0.98 |
| C1orf147 | 0.19 | 0.04 | 0.92 | RP11-689B22.2 | 0.44 | 0.21 | 0.91 |
| RP3-523K23.2 | 1.40 | 1.14 | 1.73 | RP11-302L19.3 | 0.49 | 0.25 | 0.94 |
| AC004988.1 | 0.36 | 0.14 | 0.95 | RP11-693J15.5 | 0.00 | 0.00 | 0.32 |
| RP11-359E19.2 | 0.77 | 0.63 | 0.95 | RP11-549B18.1 | 1.43 | 1.02 | 1.99 |
| CCAT1 | 1.33 | 1.02 | 1.74 | RP11-294N21.3 | 0.05 | 0.01 | 0.48 |
| LINC01031 | 0.02 | 0.00 | 0.33 | RP11-21B23.2 | 1.76 | 1.33 | 2.33 |
| RP11-304L19.13 | 0.58 | 0.34 | 0.99 | LINC00578 | 0.74 | 0.55 | 0.99 |
| RP11-218M22.1 | 0.59 | 0.41 | 0.87 | RP5-944M2.3 | 6.82 | 1.79 | 26.02 |
| RP11-116O18.1 | 0.87 | 0.75 | 1.00 | CTB-191K22.6 | 0.49 | 0.25 | 0.98 |
| CTD-3179P9.1 | 0.00 | 0.00 | 0.05 | RP11-100N20.1 | 3.93 | 1.40 | 11.04 |
| ZEB2-AS1 | 0.02 | 0.00 | 0.26 | RP11-297B17.3 | 0.09 | 0.01 | 0.57 |
| RP11-134G8.7 | 0.57 | 0.37 | 0.88 | RP11-575F12.3 | 1.65 | 1.23 | 2.20 |
| LINC00941 | 1.39 | 1.06 | 1.83 | RP3-324O17.7 | 0.26 | 0.07 | 0.92 |
| RP4-694A7.2 | 1.57 | 1.02 | 2.41 | RP11-439H9.1 | 0.02 | 0.00 | 0.85 |
| AC008984.2 | 0.00 | 0.00 | 0.63 | RP11-61G19.1 | 0.04 | 0.00 | 0.39 |
| AC104699.1 | 0.70 | 0.51 | 0.95 | RP11-269F21.3 | 1.29 | 1.01 | 1.66 |
| LINC01468 | 1.45 | 1.12 | 1.88 | LINC01312 | 9.94 | 2.91 | 34.02 |
| RP4-639F20.1 | 0.65 | 0.49 | 0.88 | CTD-2376I20.1 | 0.02 | 0.00 | 0.99 |
| RP11-108K3.2 | 2.07 | 1.12 | 3.85 | RP11-148O21.2 | 0.47 | 0.28 | 0.80 |
| AC005355.2 | 1.37 | 1.04 | 1.80 | RP11-61E11.1 | 0.12 | 0.02 | 0.75 |
| CTD-2313J17.1 | 0.41 | 0.19 | 0.90 | LINC00494 | 0.06 | 0.01 | 0.39 |
| CTD-2313J17.6 | 0.33 | 0.13 | 0.83 | AF121898.3 | 2.04 | 1.04 | 4.01 |
| LINC01268 | 0.30 | 0.10 | 0.90 | RP11-88G17.6 | 17.71 | 2.85 | 110.18 |
| RP11-739N20.2 | 1.52 | 1.04 | 2.21 | KB-1184D12.1 | 7.06 | 1.02 | 48.62 |
| RP11-768B22.2 | 0.12 | 0.02 | 0.62 | RP11-438B23.2 | 33.93 | 2.80 | 411.56 |
| MIR31HG | 1.33 | 1.04 | 1.71 | LINC01337 | 0.12 | 0.02 | 0.83 |
| LINC01150 | 0.44 | 0.25 | 0.79 | AC104113.3 | 0.07 | 0.01 | 0.63 |
| LINC01099 | 0.01 | 0.00 | 0.55 | RP11-102K13.5 | 2.14 | 1.49 | 3.08 |
| RP13-766D20.4 | 0.09 | 0.01 | 0.61 | RP11-181B11.2 | 0.01 | 0.00 | 0.34 |
| CTD-2531D15.4 | 0.27 | 0.10 | 0.72 | CASC18 | 0.13 | 0.02 | 0.96 |
| AC137932.4 | 0.50 | 0.26 | 0.96 | TMPRSS4-AS1 | 0.15 | 0.03 | 0.75 |
| RP11-383J24.1 | 1.77 | 1.11 | 2.83 | AC011899.10 | 0.09 | 0.01 | 0.96 |
| RP11-379K22.3 | 1.32 | 1.04 | 1.68 | RP11-359M6.1 | 0.69 | 0.50 | 0.95 |
| CTD-2509G16.5 | 0.20 | 0.05 | 0.84 | RP11-25K21.1 | 0.00 | 0.00 | 0.36 |
| RP11-118B22.4 | 0.45 | 0.20 | 0.98 | RP11-362K2.2 | 0.00 | 0.00 | 0.02 |
| RP11-405A12.2 | 0.56 | 0.35 | 0.91 | RP11-399H11.2 | 0.36 | 0.13 | 0.98 |
| RP11-456K23.1 | 0.44 | 0.22 | 0.90 | PRKG1-AS1 | 4.98 | 1.30 | 19.05 |
| RP1-253P7.4 | 0.32 | 0.12 | 0.81 | RP11-575F12.2 | 1.44 | 1.10 | 1.87 |
| ST3GAL4-AS1 | 1.44 | 1.05 | 1.97 | CTD-2210P24.3 | 0.01 | 0.00 | 0.50 |
| GS1-115G20.1 | 0.15 | 0.03 | 0.90 | RP11-44K6.2 | 0.77 | 0.60 | 0.99 |
| AC096579.13 | 0.51 | 0.28 | 0.91 | AC097713.4 | 0.03 | 0.00 | 0.40 |
| LINC00582 | 0.59 | 0.37 | 0.95 | RP11-388K2.1 | 3.05 | 1.04 | 8.97 |
| RP11-283G6.6 | 0.04 | 0.00 | 0.63 | CTD-2066L21.1 | 2.86 | 1.36 | 6.04 |
| RP5-981O7.2 | 0.47 | 0.23 | 0.98 | AC078852.2 | 0.05 | 0.00 | 0.55 |
| LEMD1-AS1 | 4.01 | 1.25 | 12.90 | RP11-136K7.2 | 11.54 | 1.46 | 91.48 |
| LINC00315 | 0.01 | 0.00 | 0.65 | RP5-1065O2.4 | 0.00 | 0.00 | 0.85 |
| RP11-275I4.2 | 0.08 | 0.01 | 0.68 | AJ003147.9 | 3.86 | 1.03 | 14.41 |
| RP11-1136G4.2 | 0.56 | 0.34 | 0.90 | CTD-2021H9.2 | 4.55 | 1.68 | 12.30 |
| LINC01281 | 0.24 | 0.07 | 0.85 | ELDR | 129.02 | 3.68 | 4523.03 |
| LINC00908 | 0.21 | 0.08 | 0.53 | RP3-428L16.1 | 1.50 | 1.01 | 2.21 |
| RP3-525N10.2 | 0.10 | 0.01 | 0.91 | RP11-7F17.4 | 0.00 | 0.00 | 0.16 |
| RP11-111M22.4 | 0.64 | 0.41 | 1.00 | RP5-944M2.1 | 11.20 | 1.05 | 118.99 |
| LINC01537 | 5.49 | 1.08 | 27.98 | PCAT2 | 5.59 | 1.28 | 24.40 |
| RP11-680F8.1 | 1.40 | 1.09 | 1.81 | RP11-369E15.3 | 0.00 | 0.00 | 0.42 |
| RP11-108P20.2 | 0.00 | 0.00 | 0.57 | CTD-2083E4.7 | 0.08 | 0.02 | 0.44 |
| RP11-500G10.5 | 0.35 | 0.16 | 0.77 | DEPDC1-AS1 | 70.22 | 1.63 | 3019.07 |

**Abbreviation:** ICD: immunogenic cell death; lncRNA: Long non-coding RNA; P: Probability; HR: Hazard Ratio.

**Table S3** The specific information of nine ICD-related-lncRNAs for building models after multivariate cox analysis.

| **Gene** | **Coef** | **HR** | **Se(coef)** | **Z** | **P-value** |
| --- | --- | --- | --- | --- | --- |
| CTA.384D8.34 | -0.558 | 0.573 | 0.219 | -2.550 | 0.011 |
| LINC00908 | -0.717 | 0.488 | 0.428 | -1.673 | 0.094 |
| KIAA0125 | -0.692 | 0.500 | 0.308 | -2.248 | 0.025 |
| LINC01117 | 0.968 | 2.632 | 0.305 | 3.170 | 0.002 |
| RP11.488P3.1 | 0.374 | 1.454 | 0.252 | 1.483 | 0.138 |
| RP11.114N19.3 | -0.832 | 0.435 | 0.556 | -1.495 | 0.135 |
| RP11.14N7.2 | 0.502 | 1.651 | 0.296 | 1.695 | 0.090 |
| RP11.78A19.4 | 0.517 | 1.678 | 0.304 | 1.701 | 0.089 |
| RP11.102K13.5 | 0.461 | 1.585 | 0.290 | 1.588 | 0.112 |

**Abbreviation:** ICD: immunogenic cell death; lncRNA: long non-coding RNA; Coef: Coefficient; HR: Hazard ratios; Se(coef): standard error (coefficient); Z: z scores (standard deviation); P-value: Probability.

**Table S4** Univariate and multivariate Cox regression analysis of risk factors.

| **Item** | **Univariate Cox regression** | | | |  | **Multivariate Cox regression** | | | |
| --- | --- | --- | --- | --- | --- | --- | --- | --- | --- |
|  | **HR** | **HR(95%L)** | **HR(95%H)** | **P-value** |  | **HR** | **HR(95%L)** | **HR(95%H)** | **P-value** |
| Age | 1.097 | 0.781 | 1.540 | 0.594 |  | 1.346 | 0.935 | 1.938 | 0.110 |
| Gender | 1.059 | 0.756 | 1.483 | 0.739 |  | 1.022 | 0.722 | 1.445 | 0.904 |
| Stage | 1.577 | 1.348 | 1.845 | 0.000 |  | 1.172 | 0.744 | 1.846 | 0.494 |
| T | 1.579 | 1.296 | 1.923 | 0.000 |  | 1.202 | 0.949 | 1.524 | 0.128 |
| N | 1.706 | 1.405 | 2.072 | 0.000 |  | 1.365 | 0.909 | 2.050 | 0.133 |
| M | 1.843 | 1.038 | 3.272 | 0.037 |  | 1.052 | 0.336 | 3.292 | 0.931 |
| Risk score | 1.631 | 1.449 | 1.836 | 0.000 |  | 1.567 | 1.383 | 1.775 | 0.000 |

**Abbreviation:** HR: Hazard ratios; P-value: Probability; T: Tumor; N: Node; M: Metastasis.

**Table S5** The 11 sensitive drugs in the high-risk group were obtained by chemotherapy drug sensitivity analysis at P<0.05.

| **Drug** | **P-value** | **H.median(25%,75%)** | **L.median(25%,75%)** |
| --- | --- | --- | --- |
| **Cell cycle** |  |  |  |
| BI.2536_1086 | 0.00 | 1.26(0.85-1.74) | 1.54(1.09-2.11) |
| MK.1775_1179 | 0.03 | 1.65(0.98-2.88) | 1.77(1.24-2.74) |
| **EGFR signaling** |  |  |  |
| AZD3759_1915 | 0.00 | 13.72(10.58-17.87) | 15.36(12.29-18.37) |
| Erlotinib_1168 | 0.00 | 13.06(9.52-16.51) | 15.07(11.89-18.03) |
| Gefitinib_1010 | 0.01 | 23.76(17.61-33.39) | 26.54(21.27-32.65) |
| Lapatinib_1558 | 0.01 | 18.54(12.54-27.43) | 21.81(14.71-30.84) |
| **ERK MAPK signaling** |  |  |  |
| ERK_6604_1714 | 0.00 | 26.92(19.11-45) | 33.18(24.58-45.36) |
| SCH772984_1564 | 0.00 | 12.6(6.43-24.05) | 15.4(10.24-23.62) |
| Trametinib_1372 | 0.03 | 1.53(0.73-3.53) | 1.94(1.13-3.44) |
| **Mitosis** |  |  |  |
| Docetaxel_1007 | 0.00 | 0.01(0.01-0.02) | 0.01(0.01-0.02) |
| Docetaxel_1819 | 0.03 | 0.09(0.04-0.19) | 0.11(0.06-0.22) |

**Abbreviation:** P-value: Probability; H: High; L: Low.

**Table S6** The 119 sensitive drugs in the low-risk group were obtained by chemotherapy drug sensitivity analysis at P<0.05.

| **Drug** | **P-value** | **H.median(25%,75%)** | **L.median(25%,75%)** |
| --- | --- | --- | --- |
| **ABL signaling** |  |  |  |
| Nilotinib_1013 | 0.00 | 42.14(27.69-58.91) | 32.55(21.91-48.62) |
| **Apoptosis regulation** |  |  |  |
| ABT737_1910 | 0.02 | 9.35(5.93-14.38) | 8.05(5.39-11.71) |
| AZD5991_1720 | 0.00 | 86.58(42.14-157.54) | 69.57(34.28-118.53) |
| Navitoclax_1011 | 0.00 | 7.56(4.13-13.62) | 5.92(3.5-10.57) |
| Sabutoclax_1849 | 0.00 | 0.78(0.54-1.06) | 0.59(0.42-0.75) |
| Venetoclax_1909 | 0.01 | 9.42(7.07-12.75) | 8.6(6.24-11.17) |
| **Cell cycle** |  |  |  |
| AZD5438_1401 | 0.00 | 9.79(7.03-12.85) | 8.62(6.12-11.83) |
| CDK9_5038_1709 | 0.00 | 0.1(0.07-0.15) | 0.09(0.05-0.12) |
| CDK9_5576_1708 | 0.00 | 0.73(0.53-1.04) | 0.58(0.43-0.85) |
| Palbociclib_1054 | 0.00 | 46.38(28.1-71.2) | 33.56(21.82-50.35) |
| Ribociclib_1632 | 0.00 | 46.76(40.52-56.95) | 39.43(32.87-46.86) |
| RO.3306_1052 | 0.01 | 21.2(19.42-22.56) | 20.21(18.85-22.08) |
| **Chromatin** |  |  |  |
| Entinostat_1593 | 0.00 | 9.67(7-13.28) | 8.13(6.09-11.59) |
| OF.1_1853 | 0.00 | 66.03(49.95-92.04) | 55.29(42.05-71.69) |
| PCI.34051_1621 | 0.00 | 102.66(75.24-138.09) | 77.93(57.2-110.69) |
| Vorinostat_1012 | 0.00 | 4.61(3.6-5.92) | 3.99(3.06-4.93) |
| EPZ004777_1237 | 0.00 | 187.77(138.19-239.48) | 156.32(116.74-208.78) |
| EPZ5676_1563 | 0.00 | 275.49(207.69-352.07) | 241.13(177.55-316.02) |
| GSK343_1627 | 0.00 | 17.64(15.02-21.08) | 15.55(12.69-18.15) |
| GSK591_2110 | 0.00 | 104.11(79.96-134.92) | 89.18(72.69-116.5) |
| AZD5153_1706 | 0.00 | 5.68(3.93-8.21) | 4.93(3.37-7.16) |
| I.BRD9_1928 | 0.00 | 86(63.97-116.39) | 71.7(53.87-96.3) |
| JQ1_2172 | 0.00 | 12.33(7.73-19.24) | 7.29(4.06-12.32) |
| PFI3_1620 | 0.00 | 198.19(167.67-237.54) | 183.51(146-224.74) |
| RVX.208_1625 | 0.01 | 124.44(96.39-152.94) | 110.36(85.33-141.5) |
| **Cytoskeleton** |  |  |  |
| GSK269962A_1192 | 0.00 | 18.81(15.92-24.03) | 15.43(12.59-19.14) |
| PAK_5339_1730 | 0.00 | 11.59(9.19-13.74) | 10.56(8.69-12.41) |
| **DNA replication** |  |  |  |
| Camptothecin_1003 | 0.00 | 0.1(0.06-0.19) | 0.07(0.05-0.13) |
| Cyclophosphamide_1512 | 0.00 | 182.46(142.44-231.03) | 162.5(131.87-213.03) |
| Epirubicin_1511 | 0.00 | 0.4(0.25-0.61) | 0.31(0.21-0.45) |
| Fludarabine_1813 | 0.01 | 165.86(120.02-225.74) | 145.3(104.45-196.61) |
| Gemcitabine_1190 | 0.01 | 0.57(0.25-1.54) | 0.41(0.21-0.8) |
| Irinotecan_1088 | 0.00 | 15.9(9.12-27.78) | 10.95(6.73-18.53) |
| Leflunomide_1578 | 0.00 | 156.63(126.73-198.12) | 136.09(109.02-164.92) |
| Mitoxantrone_1810 | 0.00 | 2.43(1.2-4.02) | 1.36(0.85-2.4) |
| Nelarabine_1814 | 0.00 | 433.9(328.22-572.39) | 393.02(291.77-507.7) |
| Oxaliplatin_1089 | 0.00 | 51.75(30.06-81.17) | 36.48(23.29-56.13) |
| Oxaliplatin_1806 | 0.00 | 185.43(110.94-273.02) | 121.33(83.18-189.06) |
| Pyridostatin_2044 | 0.00 | 30.69(23.8-42.18) | 26.98(19.63-36.37) |
| Teniposide_1809 | 0.00 | 1.97(1.14-3.72) | 1.32(0.72-2.24) |
| Topotecan_1808 | 0.00 | 1.27(0.73-2.42) | 0.88(0.55-1.49) |
| **ERK MAPK signaling** |  |  |  |
| KRAS(G12C)Inhibitor.12_1855 | 0.00 | 94.44(61.00-141.47) | 69.52(49.71-95.61) |
| **Genome integrity** |  |  |  |
| BIBR.1532_2043 | 0.00 | 157.94(117.85-198.31) | 135.32(101.73-172.54) |
| KU.55933_1030 | 0.04 | 77.86(63.67-100.25) | 73.74(60.4-95.39) |
| Mirin_1048 | 0.00 | 115.73(90.4-163.86) | 105.06(80.62-139.71) |
| Niraparib_1177 | 0.00 | 91.84(60.05-125.5) | 63.75(43.57-87.03) |
| NU7441_1038 | 0.01 | 13.92(12.07-16.06) | 13.11(11.17-15.66) |
| Olaparib_1017 | 0.00 | 80.41(55.99-114.83) | 66.96(47.2-91.61) |
| Telomerase Inhibitor IX_1930 | 0.01 | 1.73(1.25-2.32) | 1.52(1.2-2.12) |
| Talazoparib_1259 | 0.00 | 28.33(16.66-49.79) | 21.9(13.13-34.18) |
| **Hormone-related** |  |  |  |
| Fulvestrant_1200 | 0.01 | 19.92(14.38-24.58) | 17.27(14.14-21.87) |
| Fulvestrant_1816 | 0.00 | 101.83(72.53-132.31) | 89.91(73.06-109.69) |
| **IGF1R signaling** |  |  |  |
| BMS.754807_2171 | 0.00 | 2.26(1.81-2.8) | 1.68(1.2-2.19) |
| **JNK and p38 signaling** |  |  |  |
| Doramapimod_1042 | 0.00 | 98.79(86.64-116.14) | 81.94(70.25-95.34) |
| **Metabolism** |  |  |  |
| AGI.5198_1913 | 0.01 | 110.29(89.56-128.87) | 99.2(85.31-119.26) |
| AGI.6780_1634 | 0.00 | 66.13(51.48-86.53) | 58.75(47.51-72.1) |
| Daporinad_1248 | 0.00 | 0.01(0.01-0.02) | 0.01(0.01-0.02) |
| GSK2606414_1618 | 0.01 | 43.39(33.56-56.37) | 39.42(30.08-52.12) |
| **Mitosis** |  |  |  |
| Tozasertib_1096 | 0.00 | 20.65(15.51-25.62) | 17.23(13.4-21.77) |
| ZM447439_1050 | 0.00 | 19.28(16.38-23.96) | 16.76(13.73-20.93) |
| **p53 pathway** |  |  |  |
| MIRA.1_1931 | 0.00 | 245.08(171.71-346.8) | 202.27(147.55-286.18) |
| Nutlin-3a(-)_1047 | 0.00 | 129.52(63.38-241.68) | 81.5(47.91-152.29) |
| PRIMA.1MET_1131 | 0.00 | 121.3(85.35-175.62) | 73.4(50.87-108.49) |
| **PI3K/MTOR signaling** |  |  |  |
| Afuresertib_1912 | 0.00 | 13.57(9.25-21.05) | 10.63(7.58-15.9) |
| AMG.319_2045 | 0.00 | 137.76(106.18-187.97) | 112.71(82.83-160.89) |
| AT13148_2170 | 0.03 | 39.95(25.58-61.73) | 36.06(23.76-51.94) |
| AZD2014_1441 | 0.00 | 8.62(5.82-13.08) | 6.98(5.01-9.57) |
| AZD6482_2169 | 0.00 | 26.34(22.6-30.35) | 22.84(19.44-26.47) |
| AZD8055_1059 | 0.00 | 0.85(0.79-0.92) | 0.79(0.73-0.87) |
| AZD8186_1918 | 0.00 | 27.96(20.29-39.85) | 22.56(16.63-33.02) |
| CZC24832_1615 | 0.00 | 165.39(137.19-207.01) | 145.7(114.21-183.16) |
| Dactolisib_1057 | 0.00 | 0.22(0.15-0.32) | 0.17(0.13-0.27) |
| GNE.317_1926 | 0.00 | 1.79(1.27-2.6) | 1.5(1.11-2.18) |
| Ipatasertib_1924 | 0.00 | 34.04(25.61-52.6) | 29.11(20.97-42.39) |
| LJI308_2107 | 0.00 | 166.96(131.17-222.72) | 152.14(115.48-194.43) |
| MK.2206_1053 | 0.00 | 23.56(16.47-34.9) | 16.73(10.9-25.33) |
| PF.4708671_1129 | 0.00 | 50.26(43.53-64.6) | 41.55(35.36-52.8) |
| Pictilisib_1058 | 0.01 | 4.09(2.95-6.1) | 3.65(2.77-5.19) |
| Rapamycin_1084 | 0.00 | 0.13(0.09-0.19) | 0.1(0.07-0.15) |
| Taselisib_1561 | 0.01 | 7.62(5.04-14.38) | 6.74(4.15-11.55) |
| Uprosertib_1553 | 0.00 | 22.51(15.2-33.29) | 15.89(11.63-23.02) |
| Uprosertib_2106 | 0.00 | 19.35(11.56-31.68) | 12.97(8.86-20.72) |
| **Protein stability and degradation** | |  |  |
| ML323_1629 | 0.00 | 94.03(70.38-124.01) | 82.13(66.72-104.12) |
| P22077_1933 | 0.00 | 101.5(66.68-140.72) | 80.77(57.17-112.21) |
| **RTK signaling** |  |  |  |
| Axitinib_1021 | 0.00 | 24.3(20.03-28.07) | 19.21(15.67-23.17) |
| AZD1332_1463 | 0.01 | 50.22(36.32-69.53) | 42.81(31.44-61.94) |
| Crizotinib_1083 | 0.00 | 26.54(19.47-37.54) | 23.17(16.77-30.25) |
| PD173074_1049 | 0.00 | 62.33(43.12-93.02) | 51.53(35.42-81.11) |
| **WNT signaling** |  |  |  |
| AZ6102_2109 | 0.04 | 11.48(9.46-14.25) | 10.75(8.92-13.22) |
| IWP.2_1576 | 0.01 | 16.58(13.34-21.01) | 15.23(12.57-18.47) |
| LGK974_1598 | 0.00 | 60(41.33-83.34) | 50.86(40.08-73.07) |
| MN.64_1854 | 0.02 | 116.31(86.15-155.76) | 108.04(86.1-134.47) |
| SB216763_1025 | 0.00 | 203.35(152.37-275.95) | 144.05(107.22-207.82) |
| Wnt.C59_1622 | 0.00 | 72.06(56.55-99.59) | 60.93(50.6-77.65) |
| XAV939_1268 | 0.00 | 82.91(68.39-100.16) | 76.76(65.74-92.04) |
| **Other** |  |  |  |
| BMS.345541_1249 | 0.04 | 27.72(21.76-40.93) | 25.78(20.79-34.39) |
| Cytarabine_1006 | 0.01 | 6.01(3.28-11.96) | 4.93(3-7.88) |
| Dactinomycin_1811 | 0.00 | 0.09(0.06-0.14) | 0.08(0.05-0.11) |
| Dactinomycin_1911 | 0.03 | 0.01(0.01-0.01) | 0.01(0.01-0.01) |
| LY2109761_1852 | 0.00 | 190.56(140.68-252.3) | 165.36(117.24-220.78) |
| Picolinici.acid_1635 | 0.00 | 174.18(146.23-222.14) | 156.11(129.99-195.18) |
| TAF1_5496_1732 | 0.00 | 50.07(34.01-83.81) | 42.66(28.24-58.43) |
| Zoledronate_1802 | 0.00 | 47.6(37.16-60.54) | 39.19(29.71-51.63) |
| AZ960_1250 | 0.00 | 7.83(5.58-12.77) | 6.62(4.79-10.61) |
| AZD1208_1449 | 0.00 | 214.52(164.75-278.47) | 177.9(139.87-242.48) |
| AZD5363_1916 | 0.00 | 19.28(13.52-30.3) | 16.06(11.58-25.72) |
| Entospletinib_1630 | 0.00 | 43.48(33.62-53.81) | 36.83(29.26-52.01) |
| GSK2578215A_1927 | 0.00 | 146.39(112.25-171.84) | 127.43(106.8-156.9) |
| JAK_8517_1739 | 0.01 | 19.57(14.5-32.09) | 17.17(12.32-27.04) |
| JAK1_8709_1718 | 0.00 | 68.92(53.36-95.87) | 55.96(42.97-78.04) |
| PRT062607_1631 | 0.00 | 28.76(21.8-36.59) | 21.99(17.1-29.41) |
| Ruxolitinib_1507 | 0.00 | 137.12(104.93-170.38) | 118.69(93.27-151.92) |
| Sorafenib_1085 | 0.00 | 15.97(11.05-21.87) | 13.04(9.44-17.41) |
| WZ4003_1614 | 0.05 | 41.13(33.25-54.28) | 39.09(29.94-50.43) |
| **Unclassified** |  |  |  |
| Dihydrorotenone_1827 | 0.01 | 2.64(1.82-3.75) | 2.37(1.76-3.08) |
| Elephantin_1835 | 0.00 | 36.37(26.13-52.54) | 26.47(19.48-38.87) |
| Gallibiscoquinazole_1830 | 0.00 | 14.12(11.38-18.15) | 13.26(10.95-15.62) |
| Sinularin_1838 | 0.00 | 38.67(28.98-50.1) | 32.41(26.53-41.6) |
| Carmustine_1807 | 0.02 | 479.23(345.34-623.22) | 427.81(345.26-546.91) |

**Abbreviation:** P-value: Probability; H: High; L: Low.

**Table S7** The 68 sensitive drugs were obtained by chemotherapy drug sensitivity analysis at P>0.05.

| **Drug** | **P.value** | **H.median(25%,75%)** | **L.median(25%,75%)** |
| --- | --- | --- | --- |
| **Apoptosis regulation** |  |  |  |
| Wee1.Inhibitor_1046 | 1.00 | 7.13(4.73-12.66) | 7.25(4.78-11.02) |
| Eg5_9814_1712 | 0.99 | 0.05(0.03-0.07) | 0.04(0.03-0.07) |
| IAP_5620_1428 | 0.96 | 174.38(113.91-249.4) | 164.2(122.13-242.03) |
| ULK1_4989_1733 | 0.96 | 9.72(6.53-14.16) | 10.29(6.36-14.61) |
| Vinorelbine_2048 | 0.95 | 0.04(0.02-0.09) | 0.04(0.02-0.08) |
| PD0325901_1060 | 0.88 | 1.6(1-2.96) | 1.66(1.13-2.47) |
| Acetalax_1804 | 0.86 | 132.31(76.53-220.58) | 139.7(86.91-199.28) |
| **Cell cycle** |  |  |  |
| IGF1R_3801_1738 | 0.84 | 5.17(3.03-8.46) | 5.02(3.42-7.79) |
| Vincristine_1818 | 0.81 | 0.16(0.08-0.34) | 0.16(0.08-0.31) |
| VE821_2111 | 0.79 | 62.18(38.07-91.33) | 58.47(39.15-88.67) |
| AZD5582_1617 | 0.77 | 9.32(4.91-15.78) | 9.28(5.59-14.82) |
| **Chromatin other** |  |  |  |
| MK.8776_2046 | 0.73 | 23.31(13.79-34.77) | 23.77(13.64-38.37) |
| NVP.ADW742_1932 | 0.71 | 14.79(10.7-22.66) | 15.12(10.42-22.53) |
| OTX015_1626 | 0.71 | 11.75(8.24-17.56) | 11.09(7.81-17.47) |
| **DNA replication** |  |  |  |
| Cediranib_1922 | 0.66 | 8.45(7.1-10.81) | 8.75(6.58-10.89) |
| Foretinib_2040 | 0.66 | 2.49(1.86-3.64) | 2.58(1.9-3.68) |
| **EGFR signaling** |  |  |  |
| Ulixertinib_2047 | 0.66 | 9.11(6.62-12.63) | 9.29(7.19-12.4) |
| Podophyllotoxin.bromide_1825 | 0.65 | 0.52(0.34-0.75) | 0.53(0.36-0.74) |
| Afatinib_1032 | 0.64 | 6.06(4.14-8.99) | 5.92(4.53-7.92) |
| **ERK MAPK signaling** |  |  |  |
| WIKI4_1940 | 0.64 | 40.14(34.28-47.26) | 40.36(35.07-46.17) |
| LCL161_1557 | 0.62 | 140.47(99.74-191.33) | 143.14(109.81-173.79) |
| Alisertib_1051 | 0.56 | 6.97(3.99-12.36) | 6.78(3.72-10.8) |
| Obatoclax.Mesylate_1068 | 0.56 | 4.13(3.08-5.51) | 4.02(3.23-5.07) |
| WEHI.539_1997 | 0.50 | 35.82(22.68-51.6) | 33.41(24.16-44.2) |
| Buparlisib_1873 | 0.49 | 2.57(2.05-3.52) | 2.55(2.06-3.18) |
| VE.822_1613 | 0.49 | 29.16(16.66-48.08) | 27.26(18.7-40.67) |
| AZD4547_1786 | 0.47 | 18.21(12.01-27.74) | 17.22(12.15-27.23) |
| **Genome integrity** |  |  |  |
| Tamoxifen_1199 | 0.45 | 36.85(28.38-46.67) | 34.36(27.74-45.43) |
| Vinblastine_1004 | 0.44 | 0.02(0.01-0.04) | 0.02(0.01-0.04) |
| Pevonedistat_1529 | 0.43 | 2.15(1.17-3.53) | 1.85(1.14-3.19) |
| **Hormone-related** |  |  |  |
| Sepantronium.bromide_1941 | 0.41 | 0.01(0.01-0.02) | 0.01(0.01-0.02) |
| GDC0810_1925 | 0.40 | 142.04(107.54-183.19) | 136.15(108.38-171.37) |
| **IGF1R signaling** |  |  |  |
| Staurosporine_1034 | 0.39 | 0.04(0.03-0.07) | 0.05(0.03-0.08) |
| Selumetinib_1736 | 0.38 | 58.52(35.94-113.32) | 65.59(41.76-102.2) |
| Osimertinib_1919 | 0.36 | 5.27(3.93-7.52) | 5.59(4.25-7.36) |
| Ulixertinib_1908 | 0.36 | 16.06(10.43-24) | 16.58(11.61-22.9) |
| AZD7762_1022 | 0.35 | 1.04(0.65-1.54) | 1.03(0.73-1.58) |
| **Mitosis** |  |  |  |
| SB505124_1194 | 0.35 | 9.97(6.73-13.43) | 9.24(7.08-12.95) |
| Cisplatin_1005 | 0.34 | 26.74(15.69-41.75) | 25.51(14.21-39.22) |
| Savolitinib_1936 | 0.34 | 13.63(9.78-18.15) | 13.96(10.5-18.28) |
| Luminespib_1559 | 0.33 | 0.1(0.07-0.17) | 0.09(0.06-0.14) |
| **PI3K/MTOR signaling** |  |  |  |
| YK.4.279_1239 | 0.15 | 8.75(5.23-15.43) | 9.74(6.26-16.12) |
| Alpelisib_1560 | 0.14 | 32.79(21.84-58.17) | 30.66(21.48-49.17) |
| Paclitaxel_1080 | 0.14 | 0.06(0.03-0.12) | 0.07(0.04-0.12) |
| **Protein stability and degradation** | | |  |
| BMS.536924_1091 | 0.12 | 7.84(5.52-11.06) | 8.13(6.05-11.39) |
| Bortezomib_1191 | 0.12 | 0.01(0.01-0.01) | 0.01(0.01-0.01) |
| BPD.00008900_1998 | 0.12 | 94.23(73.26-120.37) | 87.36(67.72-115.95) |
| **RTK signaling** |  |  |  |
| Linsitinib_1510 | 0.12 | 45.6(29.84-64.38) | 42.68(29.16-58.28) |
| PLX.4720_1036 | 0.12 | 84.93(62.88-118.39) | 81.68(58.16-116.18) |
| Sapitinib_1549 | 0.12 | 48.41(32.47-72.79) | 52.93(39.92-72.26) |
| Dinaciclib_1180 | 0.10 | 0.06(0.05-0.09) | 0.06(0.04-0.08) |
| X5.Fluorouracil_1073 | 0.09 | 112.92(64.49-238.42) | 98.52(64.31-179.4) |
| Ibrutinib_1799 | 0.07 | 95.49(60.36-144.78) | 83.96(59.55-121.77) |
| **Unclassified** |  |  |  |
| IRAK4_4710_1716 | 0.07 | 144.96(114.49-179.63) | 137.27(107.9-172.64) |
| BDP.00009066_1866 | 0.06 | 10.73(8.3-14.53) | 10.05(7.77-12.79) |
| MIM1_1996 | 0.05 | 50.78(37.92-69.95) | 46.77(36.68-62.89) |
| **WNT signaling** |  |  |  |
| GSK1904529A_1093 | 0.05 | 80.61(58.43-109.57) | 73.4(58.45-94.73) |
| **Other** |  |  |  |
| MG.132_1862 | 0.29 | 0.2(0.16-0.24) | 0.19(0.16-0.23) |
| Temozolomide_1375 | 0.28 | 421.47(285.61-569.81) | 395.85(277.69-528.27) |
| I.BET.762_1624 | 0.25 | 30.11(19.45-38.65) | 26.05(19.03-40.15) |
| Dasatinib_1079 | 0.23 | 5.47(2.34-9.5) | 5.46(2.74-10.77) |
| OSI.027_1594 | 0.23 | 124.8(83.27-174.87) | 115.42(85.88-151.22) |
| UMI.77_1939 | 0.23 | 14.18(10.82-20.67) | 15.27(11.49-20.64) |
| VX.11e_2096 | 0.21 | 15.82(10.18-25.77) | 17.36(12.07-25.37) |
| Dabrafenib_1373 | 0.19 | 105.25(70.01-158.4) | 97.02(66.17-140.82) |
| VSP34_8731_1734 | 0.18 | 10.98(8.45-14.01) | 10.48(8.35-12.93) |
| AZD6738_1917 | 0.15 | 6.78(4.49-11.76) | 7.63(5.17-11.73) |
| ERK_2440_1713 | 0.15 | 12.91(8.7-21.85) | 13.83(10.32-21.2) |

**Abbreviation:** P-value: Probability; H: High; L: Low.

**Table S8** Primer sequences for nine ICD-related lncRNAs.

| **Gene** | **Primer F** | **Primer R** |
| --- | --- | --- |
| CTA.384D8.34 | TTGAGAAACGGCGTTAGGCT | GGCGTCCTCATCCCTTTTCA |
| LINC00908 | TGGCATCATCTGCCACCAAT | TTTCAAAAGCCATGCCAGCC |
| KIAA0125 | CTCCCTGCACATGATTGGGT | CCATAAGAGCACACAGCCCA |
| LINC01117 | TTCAGACAACTCTCGTGGGC | AGGCTGAATGCGAAGGAGTC |
| RP11.488P3.1 | AAGTGTGGCCAAGGGTGAAA | TTTCGGCGCCTTCCTGTTAT |
| RP11.114N19.3 | GGGGCAGCTGTCAGTCATTA | TCACCTCATGGAACTGTCGT |
| RP11.14N7.2 | GCTGGTGGGGGTCTCTTTAC | AGCAATCCTGAAACCCAGTGT |
| RP11.78A19.4 | CTTTGGGACGATGAAGGCGT | TCAAAGGGCCCTGTAACTTGT |
| RP11.102K13.5 | CATGCTGACTGCTTGTGGTG | TTCTATTTGGACGGTGGGGC |

**Abbreviation:** ICD: immunogenic cell death; lncRNA: long non-coding RNA.
